# Underwater Instant Adhesive Hydrogel Interfaces for Robust Biosensing on Diverse Species

**DOI:** 10.1002/advs.202510702

**Published:** 2025-08-24

**Authors:** Xueyin Chen, Xin Ming, Jianxiang Wang, Zenghao Xia, Xi Zhu, Lei He, Xiangyang Feng, Wenzhang Fang, Liang Huang, Zhen Xu, Yuxin Peng

**Affiliations:** ^1^ Institute of Exercise Science and Health Engineering Zhejiang University Hangzhou 310058 China; ^2^ MOE Key Laboratory of Macromolecular Synthesis and Functionalization International Research Center for X Polymers Department of Polymer Science and Engineering Zhejiang University Hangzhou 310058 China; ^3^ Department of Electrical and Electronic Engineering University of Nottingham Ningbo China Ningbo 315100 China

**Keywords:** instant adhesive interfaces, multi‐physiological monitoring, underwater biosensors, underwater hydrogel interfaces

## Abstract

Hydrogel‐assisted soft biosensors have emerged as transformative platforms for multimodal physiological monitoring in amphibious environments. However, developing instant underwater adhesive, scalable manufacturing, and robust interfaces for long‐term continuous multi‐physiological bioelectronics remains challenging. Here, a universal and facile fabrication strategy is presented that overcomes these limitations through molecular entanglement engineering to fabricate instant underwater adhesion hydrogel interfaces, exhibiting rapid, strong interfacial toughness over 230 J•m^−2^ within 10 s. Besides, a roll‐to‐roll scalable manufacturing protocol for practical transformation is established. The hydrogel interfaces with tissue‐like softness ≈109 kPa, enabling seamless multimodal biosensor integration across interfaces from aquatic organisms to amphibious species and human epidermis for reusable and long‐term usage, facilitating simultaneous multiparametric physiological signal acquisition and behavioral tracking. These findings establish a new design framework of molecular entanglement engineering for instant adhesive hydrogel interfaces, which enables reliable and rapid response biosensing technologies for healthcare monitoring and wildlife conservation.

## Graphical abstract

An instant underwater adhesion hydrogel interface is constructed via a universal and facile molecular entanglement engineering through a roll‐to‐roll scalable manufacturing protocol, achieving rapid and strong interfacial toughness over 230 J•m^−2^ within 10 s. The hydrogel interface, with a tissue‐like softness of ≈109 kPa, enables seamless biosensor integration across interfaces ranging from aquatic organisms to amphibious species and human epidermis, thereby facilitating reliable and rapid response biosensing technologies for healthcare monitoring and wildlife conservation.

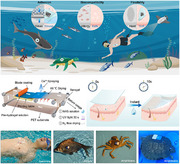

## 1 Introduction

The rapid development of flexible electronics has revolutionized conventional biosensing technologies, enabling versatile applications ranging from wearable health monitoring to crowdsensing for life entities.^[^ ^1^ ^]^ Flexible bioelectronics are beneficial for real‐time physiological monitoring during aquatic activities, including underwater sports, underwater rescue operations, and rehabilitative hydrotherapy. Furthermore, as human beings explore the underwater world gradually deeper, next‐generation underwater biosensing technologies should enable non‐invasive tracking of marine animals for obtaining organism behavior and physiological parameters to elucidate ecosystem dynamics, decipher migration patterns, and optimize human underwater locomotion.^[^ ^2^ ^]^ Meanwhile, these outdoor underwater sports and aquatic group monitoring with large‐scale operation usually require the rapid and stable deployment of biosensors to save operation time and achieve high efficiency.^[^ ^3^ ^]^

Traditional underwater monitoring systems predominantly use rigid mechanical or surgical fixation. For human health monitoring, this physical approach may restrict the target organism's movement and which most likely causes tissue damage.^[^ ^4^ ^]^ Meanwhile, suturing often leads to skin lesions and infection risks for animal tracking, undermining long‐term monitoring effectiveness.^[^ ^5^ ^]^ Although epidermal adhesive sensing devices have garnered significant attention, they still face critical technical challenges, including prolonged adhesion response time, weak device‐skin interfacial adhesion in wet environments, and non‐scalable manufacturing. These issues critically hinder their use in time‐urgent scenarios, such as aquatic biotelemetry, drowning rescue, and athletic performance monitoring. Concurrently, the conventional unimodal sensor systems are fundamentally constrained by their single data acquisition capabilities, failing to enable comprehensive multiparameter health analysis.^[^ ^6^, ^7^ ^]^ Hence, the development of multimodal biosensing systems integrated with rapidly adhesive, durable hydrogel interfaces becomes imperative for advancing both human kinesthetic evaluation and marine organism physiological monitoring. Besides, establishing standardized manufacturing protocols ensures the performance reproducibility of the bioadhesive hydrogel interface and meets the actual product transformation demands.

The fundamental mechanisms underlying insufficient adhesion strength and delayed response at device‐tissue interfaces in aquatic environments could be attributed to two primary factors: first, the reduction of interfacial molecular interactions significantly declines the hydration erosion resistance of interfacial layers.^[^ ^8^ ^]^ Moreover, unoptimized water transport channels delay the swelling kinetics of the hydrogel, thereby impeding rapid adhesion establishment.^[^ ^9^ ^]^ Recently, three main design principles have been proposed to address these challenges, including interfacial modification,^[^ ^10^, ^11^, ^12^ ^]^ nanocomposite reinforcement,^[^ ^13^, ^14^, ^15^, ^16^, ^17^ ^]^ and physical structure engineering.^[^ ^18^, ^19^, ^20^ ^]^ Specifically, the bioinspired interfacial modification strategies have demonstrated remarkable success. For instance, inspired by mussel foot proteins, tannic acid‐crosslinked chitosan and silk fibroin hydrogels form supramolecular networks via hydrogen bonding. This hydrogel achieves remarkable skin adhesion with a shear strength of 29.6kPa and adhesive hemostatic capability within 3 min for acute trauma management.^[^ ^21^ ^]^ Furthermore, the typical dual‐network interpenetrating PVA and crosslinked poly (acrylic acid) hydrogel with adhesive modification of covalently crosslinked poly (acrylic acid)‐N‐hydroxysuccinimide ester achieves rapid, non‐invasive, and stable adhesion to soft organisms with a shear strength ≈40 kPa, with not exceeding 22 s.^[^ ^22^ ^]^ For nanocomposite reinforcement strategies, incorporating 2D poly(L‐lactide)‐based nanoplatelets into calcium‐alginate hydrogels^[^ ^23^ ^]^ significantly enhances both the interfacial adhesion strength and mechanical properties of the adhesive hydrogel, achieving effective adhesion within 10 min. Currently, physical structure engineering has emerged as an advanced strategy for enhancing aquatic adhesion. For example, an ultrasonic transducer (20 kHz) triggers rapid gelation of poly(N‐isopropylacrylamide)‐alginate hydrogel on tissues via cavitation‐induced microbubble formation, which drives polymer chains into tissue microstructures, resulting in strong mechanical entanglement that enhances hydrogel adhesion, achieving ≈100 J·m^−2^ adhesion energy within 1 min.^[^ ^24^ ^]^ Despite these advances, hydrogel adhesives still suffer from slow kinetics, with conventional systems requiring over 20 s (up to 10 min) for adhesion establishment. This onset adhesive time limitation underscores the critical need for developing next‐generation underwater adhesives with ultrafast response (Table S2, Supporting Information). Overall, the developments of interface adhesion in bio‐integrated electronic systems mainly face several critical issues, including rapid adhesion kinetics, robust interfacial bonding, and scalable manufacturing processes.

Here, we employ the molecular entanglement engineering strategy (MEES) to construct underwater instant adhesive hydrogel interfaces for robust biosensing on diverse species. By leveraging the rich chain entanglements of ultra‐high molecular weight polyethylene oxide (UHMW‐PEO) incorporated with traditional polyacrylamide (PAM), the water transport channels in the prepared PEO/PAM (PAMP) hydrogels are optimized to achieve a significant enhancement in rapid swelling kinetics for instant adhesion. This PAMP hydrogel interface demonstrates ultrafast underwater adhesion within 10 s, superior interfacial bonding with interfacial toughness over 230 J·m^−2^, and tissue‐like compliance with Young's modulus ≈109 kPa for seamless and instant adhesive formation between various tissue‐device interfaces. Importantly, a roll‐to‐roll scalable manufacturing protocol is established for practical transformation. Advocated the PAMP hydrogel as a bioadhesive platform, a conformal, lightweight, and compact flexible multimodal biosensing patch enables rapid operation time within 10 s and imperceptible adhesion, facilitating long‐term, high‐fidelity monitoring of swimming activity. Furthermore, the PAMP hydrogel enables rapid and robust interfacial adhesion to both aquatic and amphibious species with biosensors, enabling reliable tracking of cross‐species underwater behavior in the field. Our findings establish an instant adhesive hydrogel platform for diverse applications in aquatic environments, like sports medicine, emergency rescue operations, and marine biological research.

## 2 Results

### 2.1 Design and Mechanism of PAMP Hydrogel for Instant Adhesive Interface

**Figure 1:**
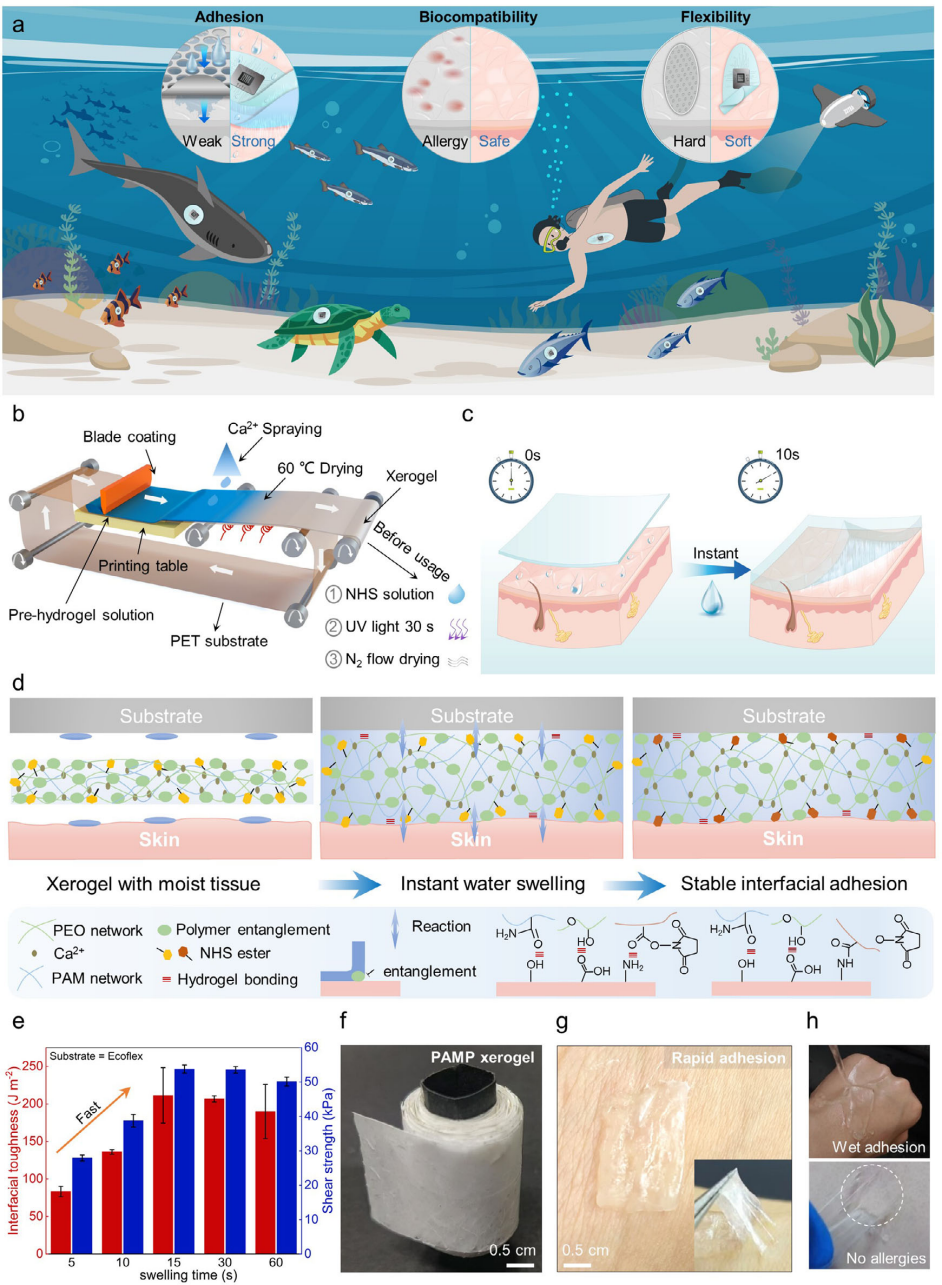
Design and mechanism of PAMP hydrogel for instant adhesive interface. a) Schematic illustration of PAMP hydrogel with strong wet adhesion, biocompatibility, and tissue‐like properties for multi‐species underwater monitoring. b) The fabrication process of PAMP hydrogel. The precursor solution was continuously transformed into PAMP xerogel. After being swollen in water for 15 s, NHS ester groups were introduced under UV irradiation, followed by nitrogen‐purged drying. c) Schematic illustration of the instant adhesion behavior of PAMP hydrogels within 10 s. d) Molecular mechanisms of the instant adhesion process of PAMP hydrogel. e) Interfacial toughness and shear strength of PAMP hydrogels with different swelling times. f) Optical photo of PAMP xerogel. g) Optical photo of a PAMP hydrogel adhered to the skin. h) Represent photos of PAMP hydrogel resisting water rinsing and undergoing nondestructive peeling.

The conventional underwater sensing devices exhibit weak adhesion to the epidermal surface, poor biocompatibility, and rigid sensor substrates that fail to achieve conformal contact with the skin. To address these limitations, we developed a tissue‐like modulus, biocompatible PAMP hydrogel via MEES to enhance the underwater adhesion performance of biosensors (**Figure** **1**a). Specifically, a uniform hydrogel precursor solution of UHMW‐PEO and PAM was blade‐coated and furtherly crosslinked with Ca^2+^ ions through a roll‐to‐roll processed PET substrate. The pre‐hydrogel on the substrate was then air‐dried at 60 °C to obtain a continuous PAMP xerogel for convenient usage (Figure 1b). The PAMP xerogel exhibits excellent transient swelling ability within 10 s to transform into the PAMP hydrogel (Figure 1c). The PAMP xerogel forms a bicontinuous porous network via polymer entanglement, which creates optimized water transport channels (Figure 1d). This unique structure significantly enhances the swelling rate, enabling instant adhesion through rapid hydration kinetics^[^ ^25^, ^26^ ^]^ (Figure S1a,b, Supporting Information). Upon contact with moist tissues of organisms, the PAMP xerogels rapidly swell within several seconds, forming adhesive bonds with skin via multiple molecular interactions to establish stable adhesion.^[^ ^27^ ^]^ Specifically, the hydroxyl groups on PEO chains and amide groups on PAM chains form hydrogen bonds with polar functional groups on the skin surface.^[^ ^28^ ^]^ The ester groups of the NHS grafted on the hydrogel surface form covalent bonds with the amine groups on the skin.^[^ ^29^ ^]^ The molecular interaction of the Ca^2+^ ion forms a coordination bond with the carbonyl oxygen in the amide group of PAM.^[^ ^30^, ^31^ ^]^ In addition, Ca^2+^ ions can also form a coordination bond with the main chain ether oxygen group of PEO.^[^ ^32^ ^]^ The synergistic effect of the two coordination bonds could contribute to enhancing the stability of PAMP hydrogels in aqueous environments^[^ ^33^ ^]^ (Figures S2 and S3, Supporting Information). Furthermore, the rich entanglements function as molecular pinning sites, significantly improving hydrogel toughness while preventing mechanical rupture under external stresses.^[^ ^34^, ^35^ ^]^

The PAMP xerogel achieves adhesion with wet skin within 10 s of contact, reaching maximum adhesion at 15 s with 90% water content, which demonstrates interfacial toughness exceeding 220 J·m^−2^ and shear strength above 53 kPa. Consequently, the PAMP hydrogel enables rapid deployment as an underwater bioelectronic adhesive layer (Figure 1e). Furthermore, the interfacial toughness reaches 108 J·m^−2^ and shear strength reaches 29 kPa at swelling equilibrium (1800 s, as shown in Figure S1, Supporting Information). This roll‐to‐roll fabricated PAMP xerogel enables rapid formation of strong skin adhesives on tissues (Figure 1f,g), exhibiting robust wet adhesion that withstands water rinsing for 30 min while allowing non‐allergenic detachment (Figure 1h; Movie S2, Supporting Information). Moreover, to evaluate the biocompatibility of the PAMP hydrogel, we performed in vitro characterization by culturing logarithmic‐phase mouse embryonic cells (NIH3T3) for 72 h on both Dulbecco's Modified Eagle Medium (DMEM) and PAMP hydrogel‐incubated media. Our results demonstrate comparable in vitro cytotoxicity of NIH3T3 cells in PAMP hydrogel‐incubated media to that of a control (DMEM) after 72 h of culture, as further confirmed by an inverted fluorescence microscope, which shows well‐preserved cell morphology (Figure S4, Supporting Information). The PAMP hydrogel meets the critical requirements for underwater applications, such as competitive athletic monitoring, emergency rescue operations, and marine biotelemetry. Significantly, it systematically addresses long‐standing challenges, including slow adhesion kinetics, weak interfacial bonding, and manufacturing scalability.

### 2.2 Swelling Behavior Tracking of PAMP Hydrogel for Instant Adhesive Interface

To elucidate the microstructural basis for rapid swelling and investigate the influence of entanglement density on the swelling properties of PAMP hydrogels, different PAMP hydrogels were prepared with a series of PAM: PEO ratios varying from 10:0 to 0:10 and PEO molecular weights ranging from 100 kDa to 8000 kDa with a PAM: PEO ratio of 5:5.

The SEM imaging reveals the distinct morphological features of different xerogels from pure PAM (10:0) to PAMP (5:5) to pure PEO (0:10). In comparison, the pure PAM xerogel demonstrated a dense and crack‐free surface, where the tightly crosslinked polymer network restricted water diffusion, slowing the swelling kinetics of the hydrogel. Whereas pure PEO xerogel collapsed into loose lamellar structures with severely fractured pore walls, reflecting insufficient structural support, which critically compromised its performance. PAMP xerogels (6:4 and 4:6) demonstrated preliminary phase separation but with markedly asymmetric domain sizes, suggesting network destabilization and further retarded swelling kinetics (Figure S5, Supporting Information).

**Figure 2:**
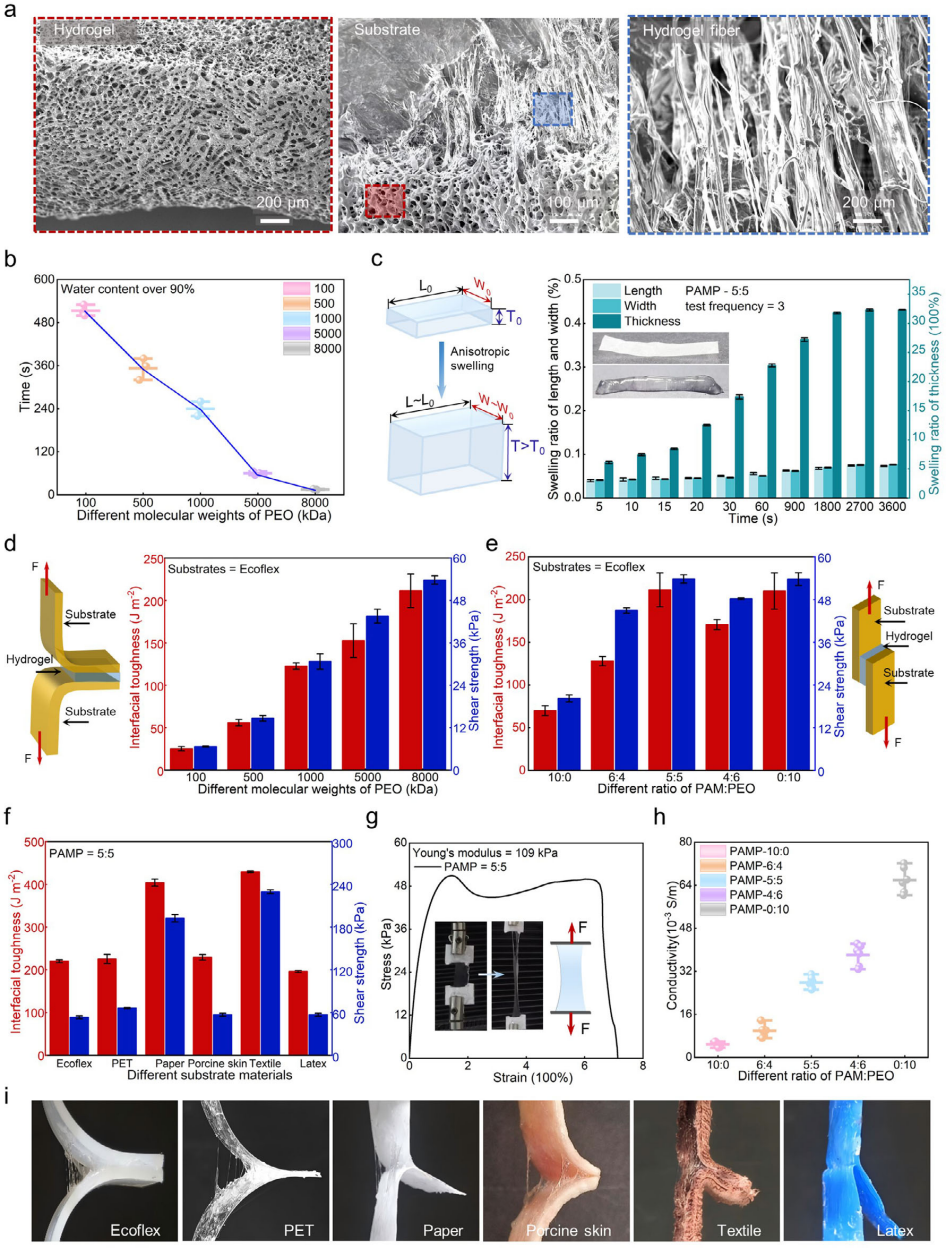
Swelling behavior tracking and interfacial adhesion regulation of PAMP hydrogel. a) SEM photos of lyophilized PAMP hydrogel, hydrogel‐substrate adhesion, and hydrogel fiber. b) Swelling time between different molecular weights of PEO from 100 kDa to 8000 kDa until the water content >90%. c) Schematic illustration for the anisotropic swelling (left) and swelling ratio in the length, width, and thickness directions of the hydrogel (right). d) Schematic illustration for maximum adhesion (left), interfacial toughness, and shear strength between different molecular weights of PEO with substrate of Ecoflex (right). e) Interfacial toughness and shear strength between different ratios of PAM and PEO with substrate of Ecoflex (left) and schematic illustration for maximum shear force (right). f) Interfacial toughness and shear strength between different substrates, Ecoflex, PET, paper, porcine skin, textile, and latex. g) Stress‐stretch curve of PAMP hydrogel. h) Electrical conductivity of different ratios of PAM and PEO. i) Representative optical photos of different substrates 180° peel test, including Ecoflex, PET, paper, porcine skin, textile, and latex.

The PAMP xerogels (5:5) and lyophilized hydrogels (5:5) showed bicontinuous interpenetrating porous networks. Moreover, the delaminated interface between PAMP hydrogel (5:5) and substrate revealed dense fibrous adhesion structures, confirming strong substrate bonding (**Figure** **2**a). These structural analyses collectively demonstrate that the PAMP hydrogel (5:5) exhibits an optimized bicontinuous porous network at the microscale. The entanglement of polymer chains provides sufficient network porosity to facilitate rapid water transport, thereby enabling fast interfacial adhesion performance. Owing to this superior architecture, the PAMP hydrogel (5:5) displays the fastest swelling kinetics, achieving over 90% water content in just 15 seconds, significantly outperforming both the PAMP hydrogels (6:4 and 4:6) (≈20 s) as well as the single‐component systems (10:0 and 0:10) (>300 s) (Figure S1a, Supporting Information).

The swelling kinetics of the hydrogels were significantly accelerated with increasing PEO molecular weight, which promoted more polymer chain entanglements (Figure S6, Supporting Information), as evidenced by the reduced time required to achieve over 90% water content. Specifically, the hydrogel with 100 kDa PEO required 500 s, whereas the 8000 kDa PEO hydrogel achieved the same swelling level in just 15 s (Figure 2b). This enhancement in swelling rate could be attributed to a more porous microstructure, providing additional water transportation channels for efficient water absorption.

The PAMP hydrogels exhibited anisotropic swelling behavior where the thickness dimension exhibited a dramatic 3200% expansion while maintaining dimensional stability in the lateral directions, making it particularly suitable for flexible patch adhesion applications (Figure 2c; Figure S7, Supporting Information). Both swelling ratio and water content progressively increased with immersion time, reaching equilibrium at 1800 s with swelling ratio exceeding 3200% and water content surpassing 96%. A remarkably rapid swelling ratio was observed, with the hydrogel achieving 700% swelling ratio within just 5s, demonstrating exceptional water absorption capacity and ultrafast swelling kinetics that satisfy the critical requirements for rapid‐adhesion biomedical applications (Figure S1b,c, Supporting Information). Moreover, to determine the lifetime of PAMP xerogels, we conducted systematic measurements of the interfacial toughness after immersion in deionised water for varying durations (with ECOFLEX substrate). The hydrogel maintained stable adhesive performance (>180 J·m^−2^) during the initial 8‐h period, followed by a gradual decline in interfacial toughness. Complete adhesive failure occurred after 35 h of immersion, with the deterioration process initiating at ≈30 h (Figures S8 and S9, Supporting Information).

The molecular interactions in PAMP hydrogels were probed through FTIR spectroscopy (Figure S10, Supporting Information). The characteristic peak at 3184 cm^−1^ gradually weakened with increasing PEO content in the binary system, while no 3184 cm^−1^ peak was observed in pure PEO hydrogel. Since this peak corresponds to the N‐H stretching vibration, the attenuation of absorption can be attributed to the formation of intermolecular hydrogen bonds between PAM and PEO. Similarly, the N─H stretching peak at 1650 cm^−1^ in pure PEO hydrogel shifted to 1660 cm^−1^ in the binary system, further confirming the influence of hydrogen bonding on the C═O stretching vibration.^[^ ^36^ ^]^ Importantly, no new characteristic peaks appeared in the binary hydrogel system, demonstrating that no other chemical bonds form between PAM and PEO, and their interaction primarily occurs through hydrogen bonding. After immersion in 6‐fluorescein solution for 2 h and removal of excess dye, the fluorescence microscopy revealed that NHS‐modified hydrogels exhibited green fluorescence, while unmodified hydrogels remained black (Figure S11, Supporting Information). This result confirmed the effective conjugation of NHS ester groups onto the hydrogel matrix to further enhance the interfacial adhesion under wet conditions.^[^ ^37^ ^]^ The PAMP hydrogel with a bicontinuous porous network through MEES facilitates swelling kinetics, thereby enabling rapid swelling and immediate adhesion to skin tissue upon contact via multiple molecular interactions.

### 2.3 Interfacial Adhesion Regulation of PAMP Hydrogel via Molecular Entanglement Engineering Strategy

The adhesive properties of PAMP hydrogels were investigated through standardized 180° peeling tests and lap shear tests, performed on hydrogels with varying molecular weight of PEO and different weight ratios of PAM/PEO. All tests were conducted using Ecoflex as a substrate. The incorporation of PEO with molecular weights ranging from 100 kDa to 8000 kDa in PAMP hydrogels demonstrated remarkable improvements in both mechanical performance and interfacial characteristics. Notably, the interfacial toughness exhibited an 8.4‐fold enhancement, escalating from 25 to 210 J·m^−2^, while the shear strength showed a 7.0‐fold increase, rising dramatically from 8 to 56 kPa (Figure 2d; Figure S12, Supporting Information). Furthermore, the interfacial adhesion exhibited a progressive enhancement with increasing PEO concentration, achieving peak performance at an optimized PAMP hydrogel (5:5), where interfacial toughness reached 230 J·m^−2^ with a 4.3 times improvement over the PAMP hydrogel (10:0) of 54 J·m^−2^. This trend was paralleled in shear properties, with strength values escalating from 20 to 56 kPa, corresponding to a 2.8‐fold enhancement (Figure 2e). When the PAMP hydrogels are peeled from the substrate, the tension propagates along the polymer chains and transfers through entanglements to neighboring chains, thereby establishing stronger intermolecular interactions at the contact interface and ultimately strengthening adhesion.^[^ ^24^, ^37^ ^]^

Standardized 180° peeling and lap‐shear tests were conducted across six substrates, Ecoflex, PET, paper, porcine skin, textile, and latex, to quantify adhesion versatility (Figure 2i; Figure Movie S1, Supporting Information). The experimental results demonstrated that PAMP hydrogel formed rapid and robust adhesion to various substrate with high interfacial toughness (>220 J·m^−2^ for Ecoflex; > 220 J·m^−2^ for PET; > 400 J·m^−2^ for paper; > 230 J·m^−2^ for porcine‐skin; >420 J·m^−2^ for textile; >190 J·m^−2^ for latex) and high shear strength (> 56 kPa for Ecoflex; > 60 kPa for PET; >190 kPa for paper; > 58 kPa for porcine‐skin; 220 kPa for textile; > 56 kPa for latex) (Figure S13, Supporting Information). Paper and textile exhibited significantly stronger adhesion, exceeding 400 J·m^−2^ and shear strength greater than 180 kPa due to their high surface roughness and abundant hydrophilic groups.^[^ ^38^ ^]^ Furthermore, Ecoflex, PET, and latex demonstrated similar interfacial toughness (>190 J·m^−2^) and shear strength (>56 kPa) (Figure 2f). Additionally, amine groups on porcine skin could react with succinimide groups on the hydrogel surface to generate NHS ester linkages,^[^ ^39^ ^]^ further strengthening the interfacial adhesion over 230 J·m^−2^ and shear strength up to 58 kPa. The interfacial toughness and shear strength between the PAMP hydrogel and tissue meet the adhesion requirements for further biosensor applications. The PAMP hydrogel exhibited outstanding cyclic stability, retaining toughness over 180 J·m^−2^ and shear strength over 49 kPa even at the ninth cycle (Figure S14, Supporting Information). PAMP hydrogels retained adhesive properties with toughness over 180 J·m^−2^ and shear strength over 49 kPa after 14‐day storage, showing exceptional reusability and storage stability. Besides, the PAMP hydrogels possessed self‐healing capacity and also adhered to diverse biomedical substrates (PET, textiles, porcine skin, Ecoflex) and rigid materials (metal forceps, pens, plastic bottles, glassware) by coating on the finger, systematically validating its superior interfacial adhesion (Figure S15, Supporting Information). We further simulated marine environmental conditions by precisely controlling temperature and hydrostatic pressure parameters, thereby broadening the potential application scenarios for PAMP hydrogels. We performed comparative 180° peel tests in an aqueous environment both before and after subjecting the hydrogel to 4 bar hydrostatic pressure for 1 h. Throughout the experiment, the water temperature was maintained at <4 °C (Figure S16, Supporting Information). Quantitative comparison of experimental results obtained before versus after pressure application showed that the interfacial toughness of the post‐pressure group (210 J·m^−2^ with substrates of Ecoflex) retained >95% of the pre‐pressure group (220 J·m^−2^) (Figure S17, Supporting Information). This demonstrates the pressure‐resistant feasibility of PAMP hydrogels, as their adhesive performance remained robust even after sustained exposure to elevated pressure.

The elongation at break of PAMP hydrogel initially increased and then decreased with increasing PEO content, while the tensile strength exhibited a gradual decline (Figure 2g; Figure S18, Supporting Information). The pure PAM hydrogels exhibited limited elongation of only 120% but the highest tensile strength of 83 kPa. In contrast, pure PEO hydrogels demonstrated superior elongation of 390% but the lowest tensile strength of 10 kPa. The elongation at break of PAMP hydrogel (5:5) reached a maximum of 700% over five times greater than that of PAMP hydrogel (10:0), while maintaining a tensile strength of 50 kPa and Young's modulus of 109 kPa. The electrical conductivity of PAMP hydrogel was measured via four‐point probe, showing an increase with PEO content. The PAMP hydrogel (5:5) achieved 3 × 10^−2^ S m^−1^, meeting the electrical conductivity requirements for flexible bioelectronics (Figure 2h). In short, the biocompatible PAMP hydrogel exhibits rapid, strong wet adhesion ability for tissue‐device interface, fulfilling critical requirements for biosensor applications like sports monitoring, emergency rescue operations, and marine biological research (Table S1, Supporting Information).

### 2.4 Instant Adhesive PAMP Hydrogel Interfaces for Swimming Activity Monitoring

**Figure 3:**
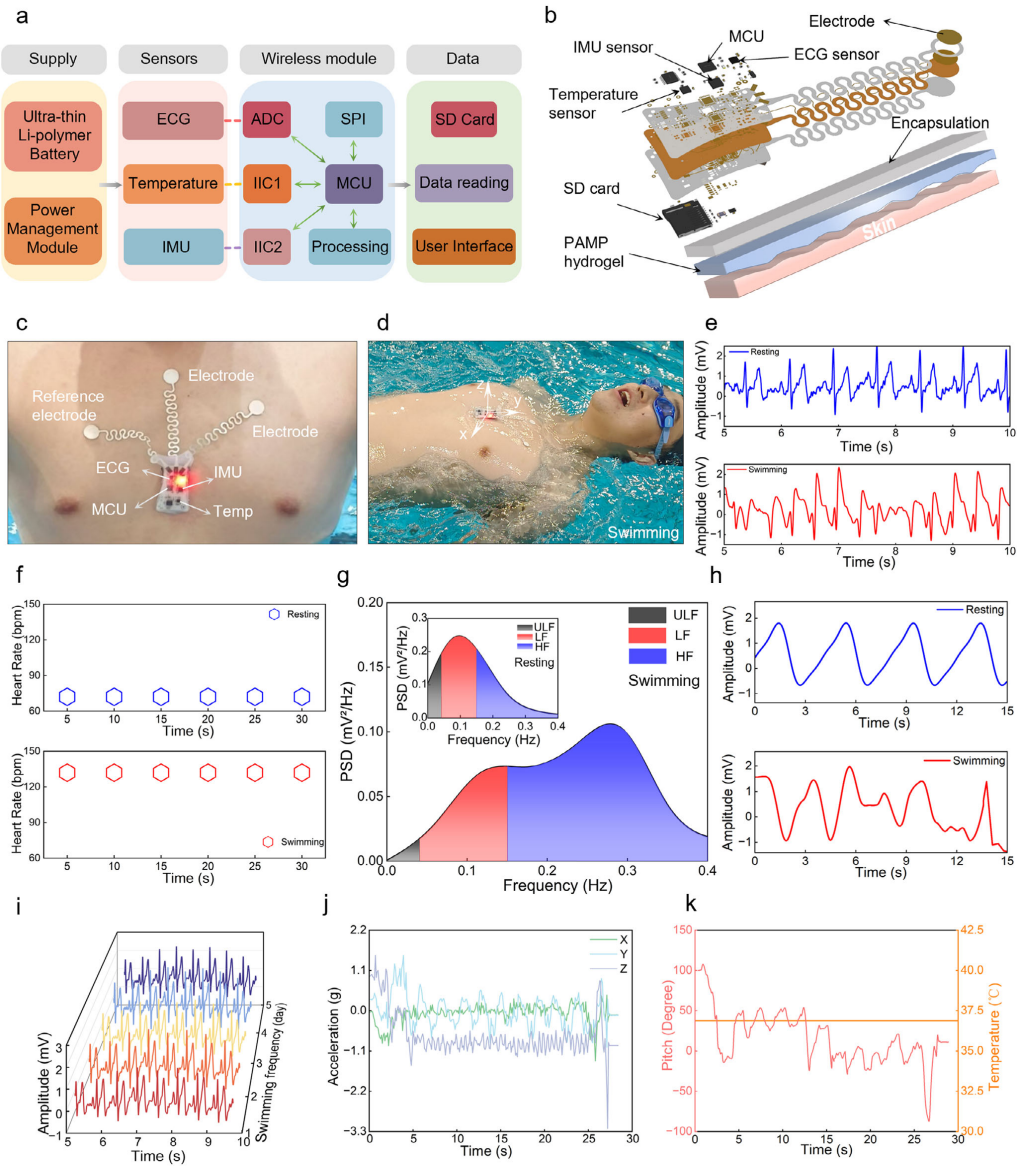
Instant adhesive PAMP hydrogel interfaces for swimming activity monitoring. a) Block diagram illustrating the key components of a flexible multimodal biosensing patch. b) Exploded‐view assembly diagram of the patch. c) Representative optical photo of the patch adhered to the chest. d) Representative optical photo of a swimmer with the attached patch. e) ECG signals of a swimmer resting and swimming detected by the PAMP hydrogel‐based patch. f–h) Heart rate, heart rate power spectral density plot, and respiration signals of a swimmer resting and swimming are calculated from ECG signals. i) ECG signals detecting capability of PAMP hydrogel‐based patch repeated use for 5 days. j) Acceleration monitoring of the swimmer detected by the patch during swimming, k) Pitch and temperature monitoring of the swimmer detected by the patch during swimming.

Conventional underwater biosensors face critical limitations like slow adhesion, single‐parameter detection, bulkiness, and poor wearability. To address these, we developed an underwater instant adhesion PAMP hydrogel interface integrated with a flexible multimodal biosensing patch (**Figure** **3**a) for synchronous monitoring of vital signs, such as heart rate, core temperature, respiratory rate, and kinematic gestures (Figure 3b). This integrated system enables rapid underwater adhesion within 10 s, minimalistic wearability, and multiparametric physiological signal acquisition during aquatic activities like swimming or diving. Specifically, through ergonomic optimization, a flexible circuit was designed to conform to pectoral muscle distribution (Figure 3c). Hybrid flexible printed circuitry with Ecoflex encapsulation ensured stable signal detection (>15 dB SNR) in dynamic aquatic environments (Figure 3d; Figure S19a, Supporting Information), making it ideal for underwater physiological monitoring during swimming, diving, and rescue operations. The system features a wireless‐powered flexible printed circuit board (FPCB) integrating ECG, IMU, and temperature sensor arrays with serpentine interconnects. Processed signals from the microcontroller unit (MCU) are stored locally on an SD card for post‐swimming wireless transfer to mobile or computer platforms (Figure S21, Supporting Information). Attaching the flexible multimodal biosensing patch to the middle of the pectoral muscle can effectively interfere with the EMG signal and reduce motion artifacts.

The underwater instant adhesive hydrogel interface ensures negligible mechanical perception during aquatic activities (Movie S3, Supporting Information). The entire 25 m lap was completed within 28 s, demonstrating consistent kinematic efficiency. Electrocardiographic (ECG) analysis revealed that the heart rate of the volunteer increased from 72 bpm (pre‐swim, SNR = 16 dB) to 132 bpm (during swimming, SNR = 17 dB), with both SNR values exceeding 15 dB, confirming signal adequacy for wearable health monitoring (Figure 3e,f). Spectral analysis of heart rate variability showed predominant low‐frequency (LF) components pre‐exercise, suggesting sympathetic activation due to test anxiety, transitioning to enhanced high‐frequency (HF) components during swimming, indicating parasympathetic dominance and stress reduction, consistent with established autonomic nervous system responses^[^ ^40^ ^]^ (Figure 3g). Furthermore, repeated monitoring over five days demonstrated excellent signal stability (Figure 3i). Respiratory signals extracted via RR interval analysis demonstrated a transition from regular 0.3 Hz breathing pre‐swim to irregular 0.4 Hz patterns during swimming^[^ ^41^ ^]^ (Figure 3h).

Acceleration analysis during the swimming trials revealed distinct biomechanical patterns across different phases of motion (Figure 3j). Upon water entry, the volunteer swimmers exhibited pronounced acceleration peaks of ≈1.5 g in both the anteroposterior (forward‐backward, y) and vertical (up‐down, z) directions, while lateral (left‐right, x) acceleration remained minimal.^[^ ^42^ ^]^ During sustained swimming, overall acceleration decreased significantly, with forward propulsion maintaining a stable magnitude of 0.3 g and vertical oscillations primarily induced by breathing actions averaging 0.1 g. Lateral acceleration remained negligible, indicating minimal side‐to‐side motion.^[^ ^43^, ^44^ ^]^ The terminal phase, characterized by flip‐turn and push‐off maneuvers at the pool wall, generated abrupt acceleration spikes, reaching 3 g vertically and 1 g anteroposteriorly. Core temperature (Figure 3k) remained stable at 37 °C during exercise, slightly elevated, potentially due to warm pool conditions or metabolic heat production.^[^ ^45^ ^]^ The flexible multimodal biosensing patch based on PAMP hydrogel successfully detected the continuous multi‐physiological signals of the swimmers, proving the reliability of the instant underwater adhesion interfaces for health monitoring.

### 2.5 Instant Adhesive PAMP Hydrogel Interfaces for Cross‐Species Behavior Detection in the Field

**Figure 4:**
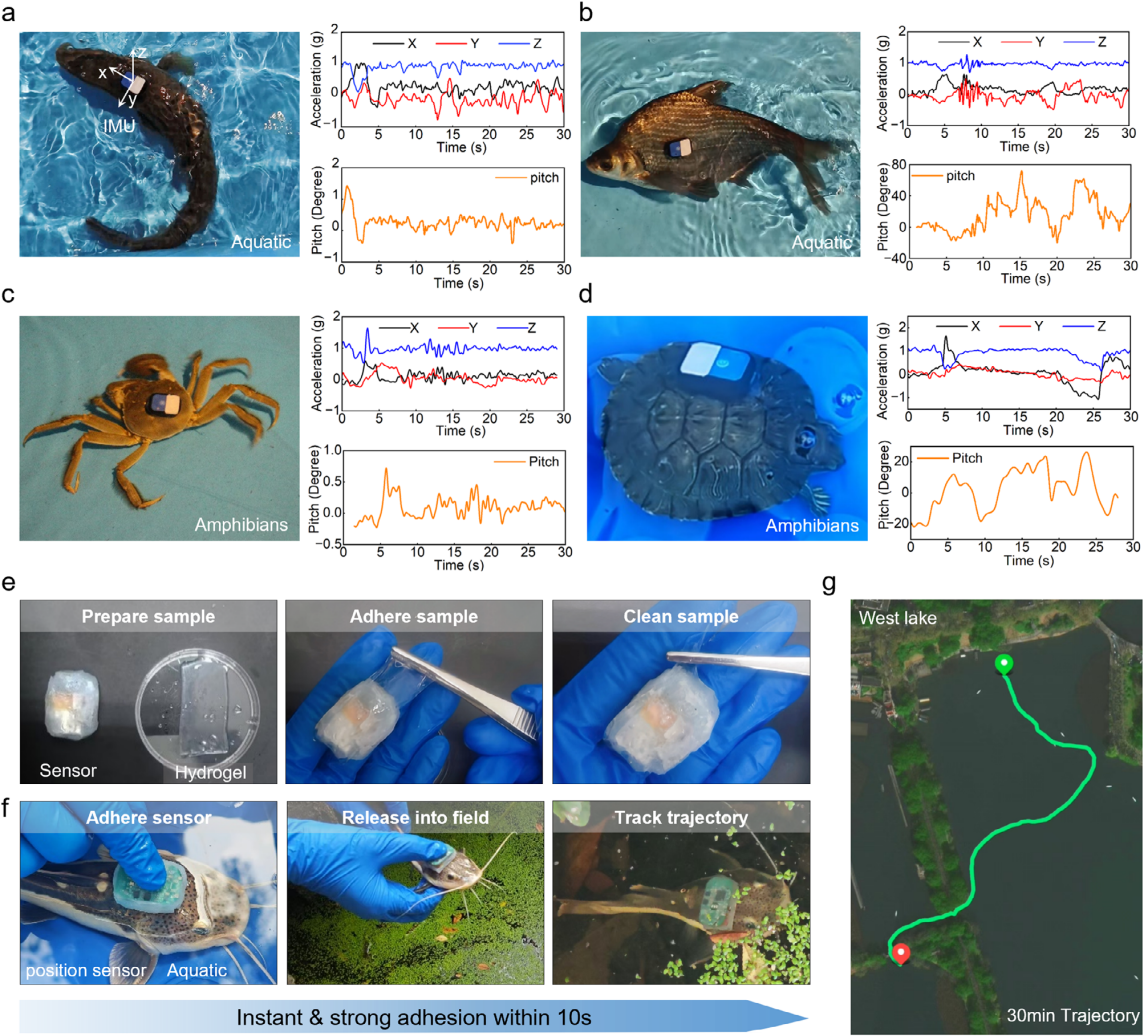
Instant adhesive PAMP hydrogel interfaces for cross‐species behavior detection in the field. a)‐d) Acceleration and pitch of aquatic and amphibian species: a) Snakehead fish, b) Bream fish, c) Crab, and d) Turtle. e) Representative photos of the PAMP hydrogel adhered to a biosensor and cleaned the sensor. f) Representative photos of the PAMP hydrogel integrated biosensor adhered to a Silurus asotus, released the fish into the field, and tracked its trajectory. g) Trajectory of the Silurus asotus in West Lake (30 mins).

Existing aquatic animal tracking methods rely on surgically sutured tags, risking tissue damage and health complications from prolonged air exposure.^[^ ^46^, ^47^, ^48^, ^49^ ^]^ Our PAMP hydrogel enables instant, non‐invasive biosensor attachment as a tissue‐compatible interfacial layer. This innovation has enabled successful monitoring and trajectory tracking of ecologically key aquatic species, like blackfish (**Figure** **4**a), bream (Figure 4b), amphibious creatures, crabs (Figure 4c), and turtles (Figure 4d). Consistent attachment was achieved within 10 s per specimen using the standardized protocol (Movie S4, Supporting Information). All animals were ethically sourced from local agricultural markets and scheduled for release to protected aquatic habitats post‐experimentation. The PAMP hydrogel integrated biosensor demonstrated reliable 48‐h integration across species owing to its tissue‐mimetic compliance and robust wet adhesion, enabling long‐term behavioral monitoring without compromising animal welfare (Figure S19b, Supporting Information). Crucially, the biosensor withstood dynamic challenges, including rapid vertical oscillations of bream with peak acceleration of 1 g and crab flipping maneuvers with peak acceleration of 1.5 g, validating exceptional adhesion stability under complex biomechanical loading.^[^ ^50^ ^]^

To validate the field efficacy of PAMP hydrogel, we deployed the PAMP hydrogel integrated biosensor on a Silurus asotus for trajectory tracking, where the biosensor was gently pressed onto the dorsal surface and the fish was immediately released into West Lake, Hangzhou, China (Figure 4f). Moreover, the contact interface between the biosensor and the Silurus asotus skin consists of a biocompatible PAMP hydrogel, ensuring no adverse effects on the epidermis. The field‐deployed biosensor weighs less than 8 g, imposing negligible additional pressure and minimal mechanical stress on the Silurus asotus during monitoring.

The entire attachment process was completed within 10 s by a single operator (Movie S5, Supporting Information). For this field trial, the biosensor was fabricated under laboratory conditions and transported in hydrophobic‐coated Petri dishes before deployment (Figure 4e). Post‐deployment tracking through radio‐frequency telemetric monitoring captured continuous high‐resolution swimming trajectories with remarkable stability for sustained periods exceeding 30 min (Figure 4g). Concurrently, the PAMP hydrogel maintained uninterrupted adhesion integrity throughout 24‐h monitoring cycles, as established in controlled aqueous environment trials (Figures S19b and S20, Supporting Information).

This cross‐species behavior detection demonstrates the instant underwater adhesion capability of PAMP hydrogel for suture‐free monitoring of aquatic species, opening new possibilities for conservation biology applications, particularly for endangered species like sea turtles and Chinese sturgeons.

## 3 Conclusion 

Our work establishes a new design in bio‐adhesive technology through molecular entanglement engineering, demonstrating a universal strategy for fabricating instant underwater‐adhesive hydrogel interfaces with exceptional interfacial toughness (>230 J·m^−2^), tissue‐like compliance (109 kPa), and roll‐to‐roll manufacturability. By constructing polymer entanglement engineering, the PAMP hydrogel overcomes fundamental limitations in aquatic biosensing, achieving 10s adhesion kinetics and seamless integration with multimodal patches and biosensors for simultaneous monitoring of human health and aquatic tracking. This work overcomes the long‐standing challenges in wet adhesive hydrogels, including prolonged adhesion response time, weak device‐skin interfacial adhesion in wet environments, and non‐scalable manufacturing. These advances create new standards for next‐generation bio‐interfaces in aquatic sports medicine, wildlife conservation, and emergency rescue, while providing a generalizable molecular design framework for adaptive hydrogel materials in extreme environments.

## Conflict of Interest

The authors declare no competing interests.

## Author Contributions

X.C. and X.M. contributed equally to this work. Y.P., X.M., Z.X., and X.C. conceived conceptualization; X.C. and X.M. performed methodology; X.C., X.M., J.W., Z.X., X.Z., L.H., X.F., and L.H. performed investigation; X.C., X.M., and Y.P. performed writing‐original draft; X.C., X.M., Z.X., and Y.P. performed writing‐review & editing; Y.P. and Z.X.acquired funding acquisition. All authors participated in the discussion and provided comments on the paper.

## Ethical Statement

The experiments adhered to ethical guidelines. All experimental animals, including fish, turtles, and crabs, were purchased from local markets. After the experiments, the fish were released into safe and natural environments. The study protocol was approved by the Medical Ethics Committee of the Department of Psychology and Behavioral Sciences, Zhejiang University, China (reference number: 2023[091]).

## Supporting information



Supporting Information

Supplemental Movie 1

Supplemental Movie 2

Supplemental Movie 3

Supplemental Movie 4

Supplemental Movie 5

## Data Availability

The data that support the findings of this study are available from the corresponding author upon reasonable request.
