# Supplementary material for: Underwater Instant Adhesive Hydrogel Interfaces for Robust Biosensing on Diverse Species
Source: Adv Sci (Weinh). 2025 Aug 24;12(43):e10702. doi: 10.1002/advs.202510702 (PMC12631874; doi:10.1002/advs.202510702)
Supplement: Supplementary file 1 — Supporting Information [file ADVS-12-e10702-s001.pdf]

Supporting Information

Underwater Instant Adhesive Hydrogel Interfaces for Robust Biosensing on Diverse Species

Xueyin Chen[1+,3], Xin Ming[2+], Jianxiang Wang[1], Zenghao Xia[1], Xi Zhu[1], Lei He[2], Xiangyang Feng[2], Wenzhang Fang[2], Liang Huang[3], Zhen Xu[2], Yuxin Peng[1*]*

[1]Institute of Exercise Science and Health Engineering, Zhejiang University, Hangzhou 310058, China

[2]MOE Key Laboratory of Macromolecular Synthesis and Functionalization, International Research Centre for X Polymers, Department of Polymer Science and Engineering, Zhejiang University, Hangzhou 310058, China

[3]Department of Electrical and Electronic Engineering, University of Nottingham Ningbo China, Ningbo 315100, China

[#]These authors contributed equally to this work.

*Corresponding author. Email: xin_ming@zju.edu.cn; yxpeng@zju.edu.cn

This file includes:

Methods

Supplementary Figures 1 to 21

Supplementary Tables 1 to 2

Supplementary References

Other Supplementary Materials for this manuscript include:

Supplementary Movies 1 to 5

Methods

Materials

Polyacrylamide, Polyethylene oxide, $CaCl_2$, Dimethyl sulfoxide, N-acryloxysuccinimide 6-aminofluorescein, ethanol, Ecoflex, and benzophenone were purchased from Aladdin and used. The other reagents were used directly upon receipt without further processing.

Characterization

The structure and morphology of PAMP hydrogel were investigated by SEM (Hitachi S4800) and fluorescence microscope (KEYENCE VK-X150). The molecular consistent of PAMP hydrogel was determined by infrared spectroscopy (Nicolet is 10). The shear, peel, and stretch tests were conducted by a mechanical machine (Instron Legend 2344). The electrical conductivity of PAMP hydrogel was measured by a digital source meter through the four-probe method (Keithley 2400). Hydrostatic pressure tests were conducted on the pressure valve, which was developed by the Ocean Technology and Equipment Centre, Hangzhou Dianzi University, China. In vitro characterization was performed by an inverted fluorescence microscope (Axio Vert.A1) and Dulbecco's Modified Eagle Medium (10-013-CVRC).

Scalable preparation of the PAMP hydrogel

A uniform pre-solution of hydrogel (2 mm thick) was blade-coated onto a rolled PET substrate (treated with a hydrophobic coating). Ca^{2+} ions were sprayed onto the polymer surface uniformly. The coated substrate was then air-dried at 60°C to obtain a continuous PAMP xerogel. To introduce the NHS ester network, the PAMP xerogel was cut into desired dimensions, swollen in water for 15 seconds to achieve a water content of 90%, and subsequently immersed in an anhydrous DMSO solution containing 2 mg/mL NHS ester and 0.2 wt% benzophenone. The hydrogel was then exposed to UV light (365 nm) for 30 seconds to complete the crosslinking reaction. Finally, the PAMP hydrogel was dried under nitrogen flow (N_2 flow). PAMP xerogels rapidly swell within several seconds, forming adhesive bonds with skin via multiple molecular interactions to establish stable adhesion.

Swelling ratio and water Content

To measure the swelling ratio and water content of the hydrogel, hydrogel samples (15 mm × 5 mm) were immersed in water and a 3.6%wt NaCl solution for swelling under constant temperature conditions (25 ± 0.5°C). The dimensional changes in three dimensions were

measured using a micrometer, and three independent experimental trials were performed. The swelling ratio (SR) was calculated according to Equation (1):

$$\mathrm{SR_i} = (\mathrm{D_s} - \mathrm{D_0})/\mathrm{D_0} * 100\% \ (\mathrm{i} = \mathrm{x}, \mathrm{y}, \mathrm{x}) \qquad \ldots\ldots (1)$$

where D_0 and Ds represent the dimensions before and after swelling, respectively. The overall swelling ratio (SR) of the hydrogel was calculated according to Equation (2):

$$SR = \left((\mathrm{m_s} - \mathrm{m_d})/\mathrm{m_d}\right) * 100\% \qquad \ldots\ldots (2)$$

where m_d and m_s denote the mass of the hydrogel in the dry and swollen states, respectively. The water content (WC) was calculated according to Equation (3):

$$\mathrm{WC} = \left((\mathrm{m_s} - \mathrm{m_d})/\mathrm{m_s}\right) * 100\% \qquad \ldots\ldots (3)$$

where m_d and m_s denote the mass of the hydrogel in the dry and swollen states, respectively.

Electrical conductivity testing

The electrical conductivity (σ) of the hydrogel was measured using a four-probe method; three independent experimental trials were performed, and the electrical conductivity was calculated using the following formula (4):

$$\sigma = \left(\mathrm{L}/(\mathrm{R} * \mathrm{A})\right) \qquad \ldots\ldots (4)$$

where L is the electrode spacing, A is the effective conductive area, and R is the measured resistance.

Mechanical tests

The interfacial toughness and shear strength were systematically evaluated through standardized 180° peel tests and lap shear tests conducted in compliance with ASTM standards. All mechanical tests were performed using an electronic universal testing machine at a constant speed of 100 mm/min. For consistency, specimens were prepared under controlled conditions with substrate dimensions of 2 cm × 0.5 cm and hydrogel samples precisely trimmed to 1.5 cm × 0.5 cm rectangles. Before adhesion testing, PAMP xerogel was equilibrated to 90% water content through controlled swelling. Sample preparation involved applying a uniform pressure of 1 kPa for 30 seconds using either the testing machine's compression mode or calibrated weights to ensure proper interfacial contact. To eliminate ageing effects, all tests were initiated immediately after specimen preparation. Interfacial toughness was calculated by dividing the plateau peak force from the 180° peel test by the width of the tissue sample, while shear strength was determined by dividing the maximum force by the adhesion area. The adhesion performance was comprehensively characterized across multiple substrates, including textile, Ecoflex, PET, paper, latex, and porcine skin. Parallel tensile testing under identical

displacement rate conditions (100 mm/min) enabled the determination of Young's modulus from the linear elastic region of stress-strain curves. All reported mechanical properties represent the mean of at least three replicate measurements per condition. PET and Ecoflex substrates were pretreated with a commercial silicone-based primer for surface modification, while paper and textile were tested without additional treatment; fresh porcine skin was gently wiped with a lint-free cloth to remove surface lipids, and latex underwent ultrasonic cleaning for complete degreasing before adhesion testing.

Integrated with a multimodal biosensing patch

The flexible multimodal biosensing patch was encapsulated using silicone elastomer. Ecoflex was prepared by thoroughly mixing the base and curing agent for 3 minutes, followed by 1 minute of vacuum degassing to remove air bubbles. The prepared mixture was then cast into a custom-designed mold featuring a central cavity to accommodate the sensor module, and cured at ambient temperature (25±2°C) for 1 hour to achieve optimal mechanical properties. Before sensor integration, the cured silicone surface was treated with a proprietary silicone primer to enhance adhesion. The primer was uniformly applied and allowed to air-dry for 10 minutes under controlled laboratory conditions. For final assembly, the hydrogel sensing element was directly bonded to the primed silicone surface through conformal contact, ensuring continuous interfacial coupling during physiological monitoring applications.

Human monitoring

The circuit schematic and PCB layout were designed and finalized. The microcontroller (MCU) control program was developed using the Arduino integrated development environment (IDE). Data processing and analysis were performed using Python scripts. On October 27, 2024, the flexible multimodal biosensing patch was assembled in the laboratory and subsequently deployed for testing in the swimming pool at Zhejiang University. During testing, the flexible multimodal biosensing patch was securely attached to the volunteer's chest region using PAMP hydrogel. Physiological parameters were successfully recorded throughout a complete 25-meter swimming session. To validate system reliability, continuous data acquisition was conducted over five consecutive days. This testing regimen confirmed both the repeatable performance of the sensing patch and its operational stability in underwater wet conditions. Between test sessions, the sensor was carefully cleaned and stored to maintain its functional integrity for subsequent deployments.

Diverse Species monitoring

To evaluate the effectiveness of hydrogel biosensors in adhesion across various species interfaces, two inflatable animal pools were purchased, one measuring 120 × 90 × 36 cm and the other 200 × 150 × 60 cm. Multiple tests were conducted between September 25, 2024, and November 10, 2024. Freshwater was used for the experiments and was exposed to sunlight for 8 hours before use to allow the animals to acclimate. Experimental animals, including fish, turtles, and crabs, were sourced from a local market near the laboratory and were released into nearby safe water bodies after use.

In this study, two sensors were employed, a three-axis inertial measurement unit (IMU) with an accelerometer, gyroscope, and magnetometer sampling at 200 Hz, and a GPS for trajectory tracking. Animals were quickly removed from the water, and hydrogel adhesive was applied to attach the sensors within 10 s. All animals were immediately released back into the water after sensor attachment. The experimental animals included a cuttlefish (50 cm in length), a bream fish (20 cm in length, 10 cm in width), a turtle (10 cm in length), and a crab (8 cm in carapace width).

We evaluated the field performance of PAMP hydrogel in natural aquatic environments through an experiment conducted at West Lake, China, on November 9, 2024. A GPS sensor was rapidly adhered to a Silurus asotus using PAMP hydrogel within 10 seconds, followed by immediate release into the lake. The swimming trajectory was continuously monitored for 30 minutes under natural hydrodynamic conditions, demonstrating the effectiveness of PAMP hydrogel for aquatic biosensor application.

Multi-physiological signal processing

The statistical significance of all comparative experiments in this study was evaluated using Origin software. Unless otherwise specified, the sample size for all experiments was three. Calculations of water content and swelling ratio were performed using Excel software. Signal-to-noise ratio (SNR), heart rate, heart rate power spectral density, and respiratory signals were computed using Python software.

Since the energy of the ECG signal is primarily concentrated in the range of 0.5 Hz to 40 Hz, while noise mainly originates from power frequency interference and high-frequency noise from electromyographic signals (above 40 Hz), the ECG signal power and noise power were obtained by integrating the signal and noise frequency bands, respectively. The SNR is calculated using the following formula (5):

$$\mathrm{SNR\ (dB)}=10\cdot\log_{10}\left(\frac{P_{signal}}{P_{noise}}\right) \qquad (5)$$

where P_{signal} is the signal power and P_{noise} is the noise power. ECG signal noise is categorized into three levels: For clinical diagnostic-grade ECG signals, the SNR typically needs to be ≥ 20 dB, with clear visualization of P waves, QRS complexes, and ST segments (AAMI EC11 standard). For medical-grade dynamic monitoring ECG signals, the SNR generally ranges between 15–20 dB, ensuring reliable heart rate detection and arrhythmia analysis. For wearable devices, the SNR should be within 10–15 dB, enabling heart rate monitoring[8]-[10].

Heart rate was calculated using the RR interval method[11]. The RR interval (R-R Interval) refers to the time interval between two consecutive R waves in the ECG signal, calculated using the following formula (6):

$$\text{Heart rate}=\frac{60}{\text{R-R Interval}} \qquad (6)$$

where Heart rate is expressed in beats per minute (bpm), and the R-R Interval is in seconds (s).

Heart rate power spectral density (PSD)[12] is a frequency-domain analysis method for heart rate variability (HRV), quantifying the energy distribution of cardiac rhythms across different frequencies. It reflects the regulatory effects of the autonomic nervous system (sympathetic and parasympathetic) on the heart and serves as an essential tool for assessing cardiovascular health and neural regulation. The heart rate PSD is divided into three frequency bands: ULF (<0.04 Hz), the Ultra-low frequency band, associated with circadian rhythms, thermoregulation, and hormonal secretion (e.g., cortisol, melatonin). LF (0.04 Hz–0.15 Hz), Low-frequency band, related to baroreflex (e.g., changes in body posture or mental stress) and sympathetic activity, potentially reflecting the combined effects of sympathetic and parasympathetic systems. An increase in LF power indicates heightened sympathetic activity, such as anxiety[13][14]. HF (0.15 Hz–0.4 Hz), High-frequency band, linked to respiratory activity and reflecting rapid vagal (parasympathetic) modulation of heart rate. A decrease in HF power indicates reduced vagal tone, such as chronic stress, while an increase in HF power can assess cardiac rehabilitation effects[15], such as enhanced parasympathetic activity after exercise training. The PSD was computed using the Fourier transform.

Respiratory signals during exercise were extracted directly from the ECG signal using the RR interval method. This is because respiration modulates heart rhythm through the nerve, causing RR intervals to shorten during inhalation and lengthen during exhalation. The fluctuation frequency of RR intervals is synchronized with the respiratory frequency (typically 0.1–0.5 Hz, or 6–30 breaths per minute).

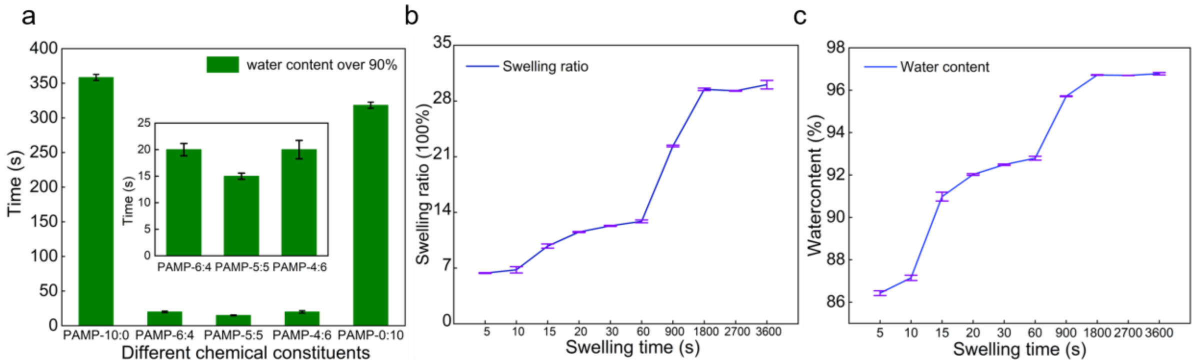

Figure S1. Swelling dynamics of PAMP hydrogel. a) Comparison of the time required to reach 90% water content across different PAMP hydrogel compositions. b) Swelling ratio as a function of time illustrating the PAMP xerogel swelling kinetics. c) Water content as a function of time illustrating the PAMP xerogel swelling kinetics.

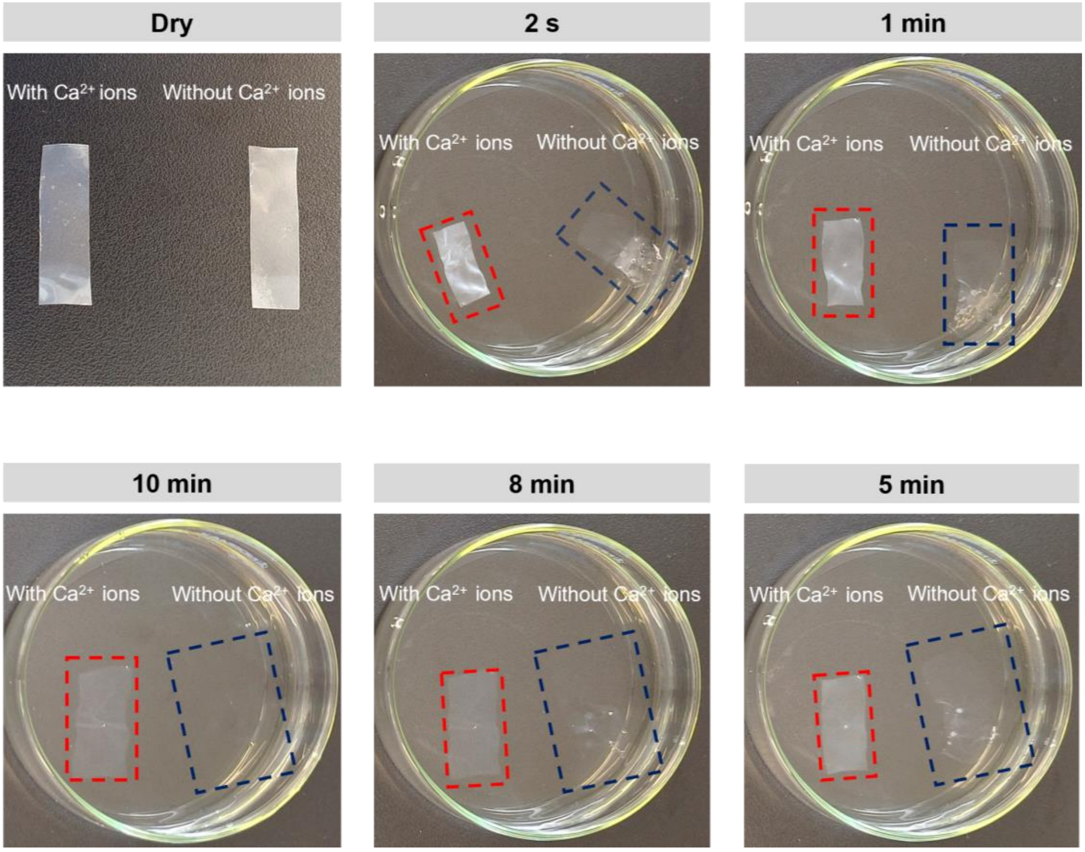

Figure S2. Representative images comparing the dissolution of PAMP xerogel samples with and without Ca^{2+} ions.

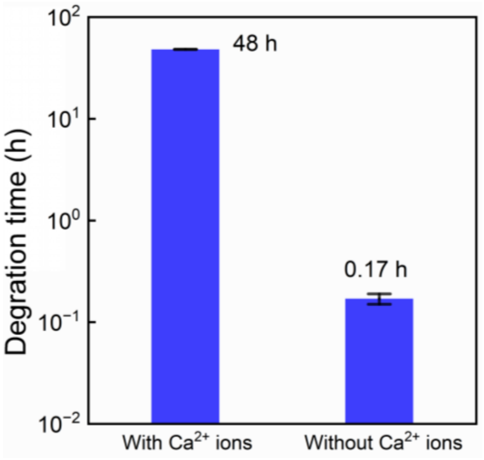

Figure S3. Comparison of complete dissolution times for PAMP xerogel samples in deionized water with and without Ca^{2+} ions (n=3).

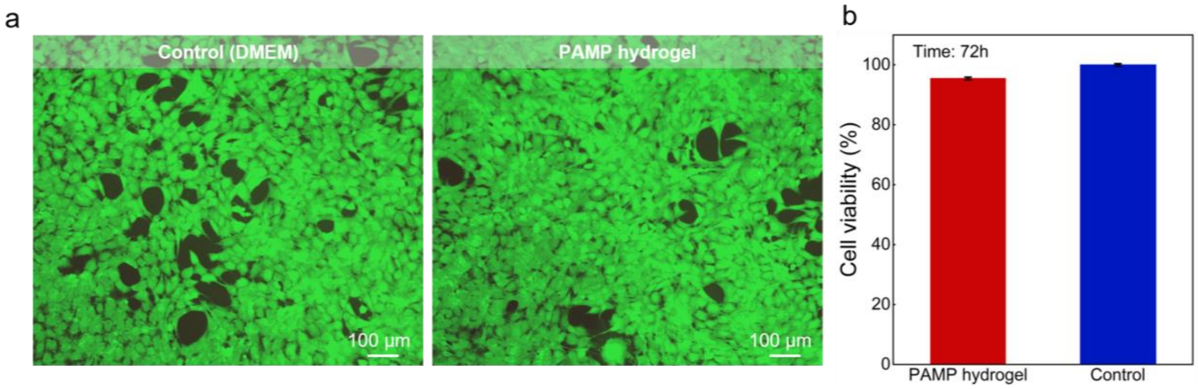

Figure S4. In vitro biocompatibility of the PAMP hydrogel. a) Representative inverted fluorescence microscope images of Live/Dead assay of logarithmic-phase mouse embryonic cells (NIH3T3) after 72-hour culture in the control (Dulbecco's Modified Eagle Medium, DMEM) and the PAMP hydrogel-incubated Medium (n=3). b) In vitro cell viability of logarithmic-phase mouse embryonic cells based on Live/Dead assay after 72 h of culture in a control medium (DMEM) and PAMP hydrogel-incubated Medium.

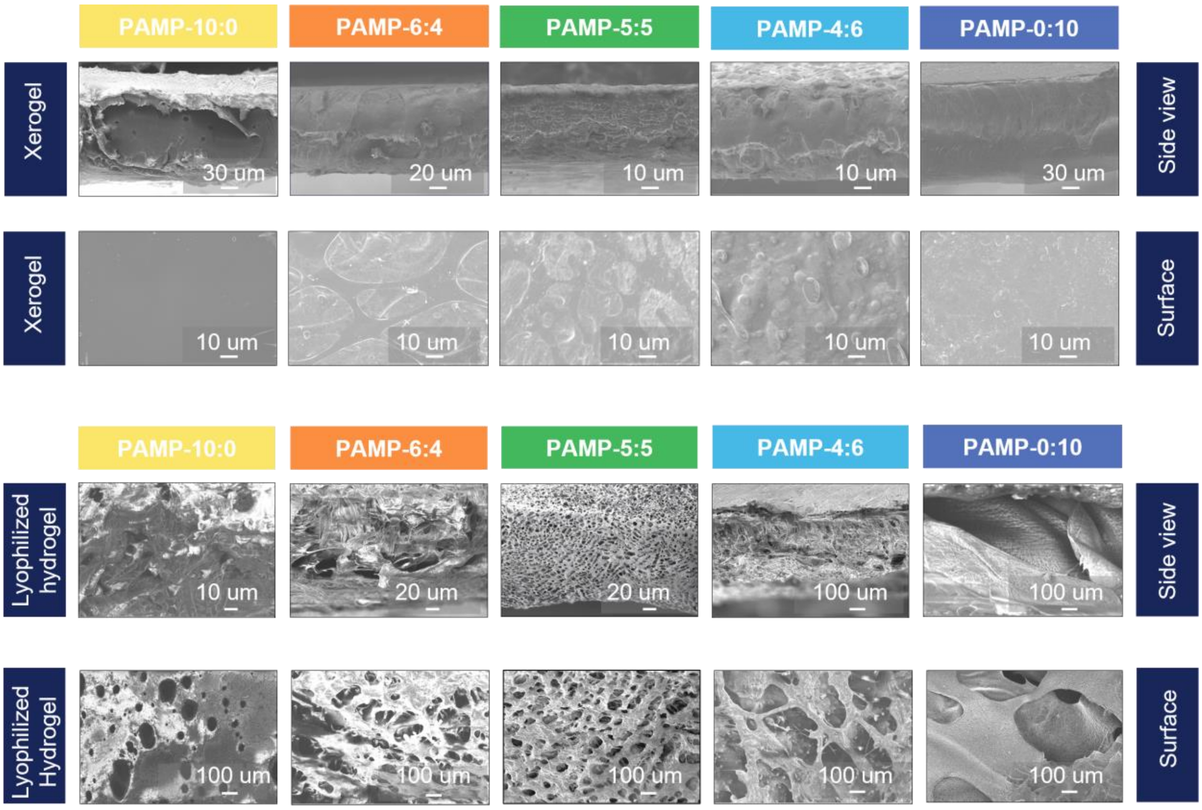

Figure S5. SEM images of the side view and surface of xerogel and lyophilized hydrogel with different chemical constituents of PAMP.

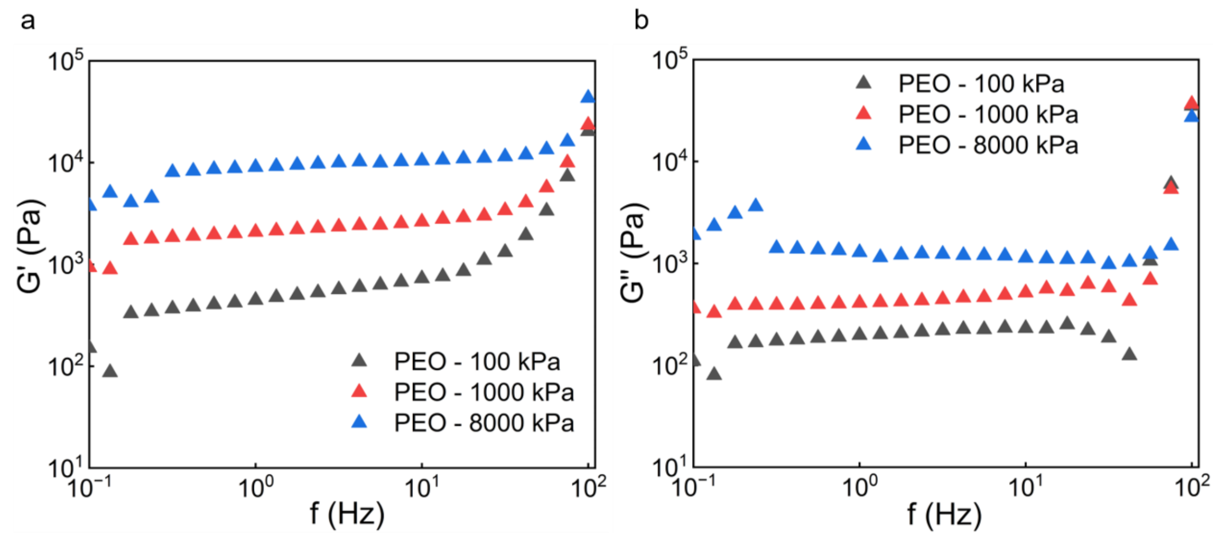

Figure S6. Mechanical characterization of PAMP hydrogels with different molecular weights of PEO. a) Frequency dependence of storage modulus (G'). b) Loss modulus (G'').

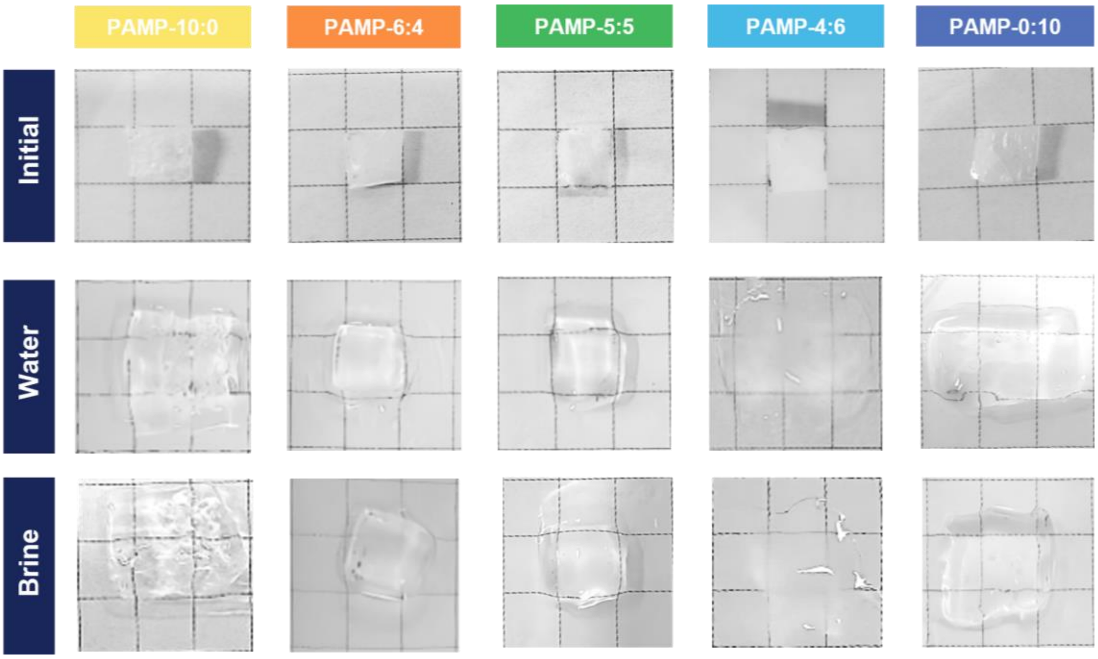

Figure S7. Representative appearance of PAMP hydrogels with different material compositions after full swelling in deionized water and 3.6 wt% brine solution with stability. Scale bar: 5 mm (each grid division).

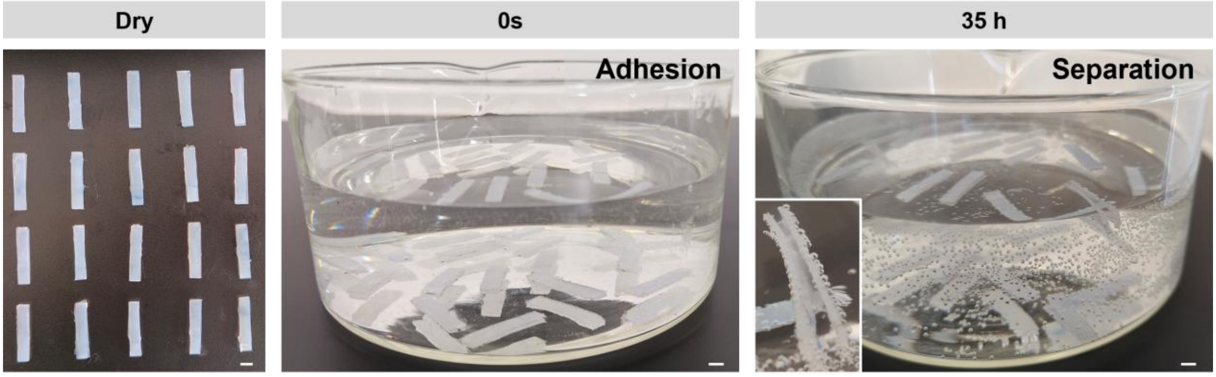

Figure S8. Representative images of the time-dependent morphological evolution of PAMP xerogel specimens adhered to Ecoflex substrates under continuous deionized water immersion, the interfacial bubbles originate from capillary effects induced by localized hydrophobic regions on the Ecoflex surface. Scale bar: 0.5 cm.

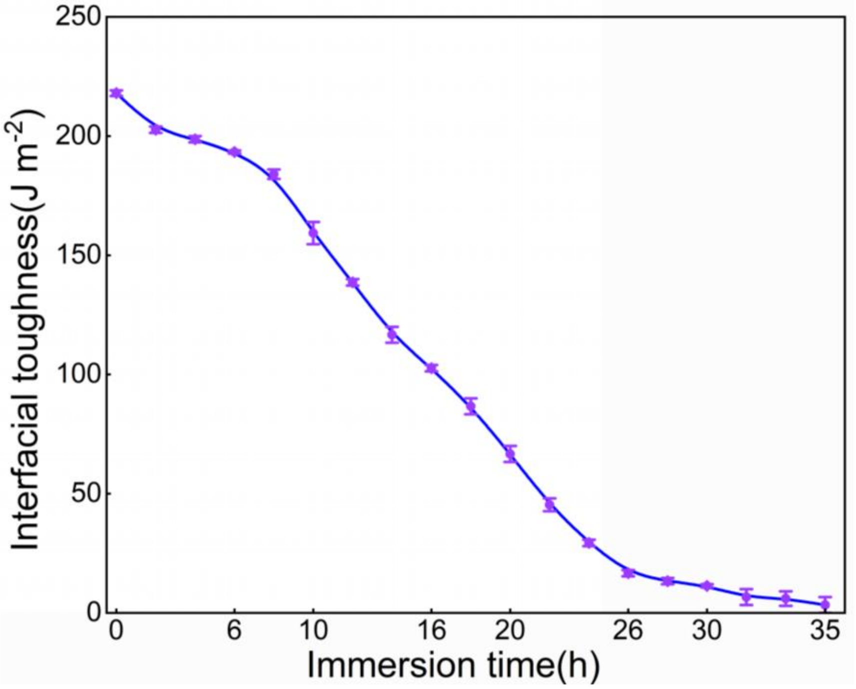

Figure S9. The interfacial toughness after immersion in deionized water for varying durations (with Ecoflex substrate) (n=3). Periodic 180° peel tests were conducted during continuous immersion in deionized water.

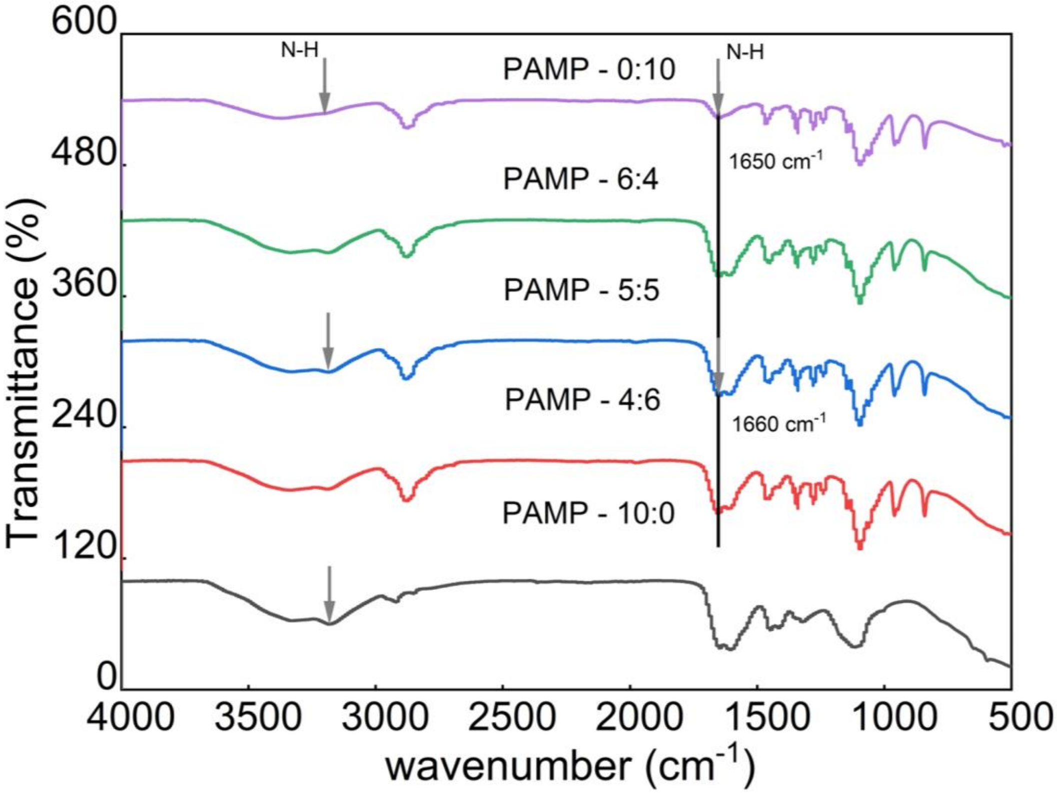

Figure S10. FTIR spectra of PAMP hydrogels with different chemical compositions.

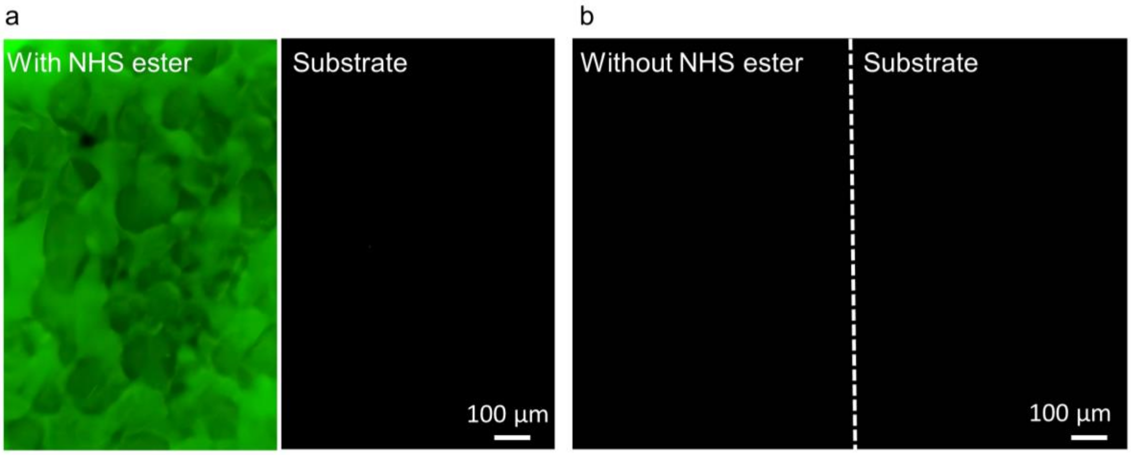

Figure S11. Characterization of PAMP hydrogel. a) Fluorescence microscopic images of PAMP hydrogel with NHS ester. b) Fluorescence microscopic images of PAMP hydrogel without NHS ester.

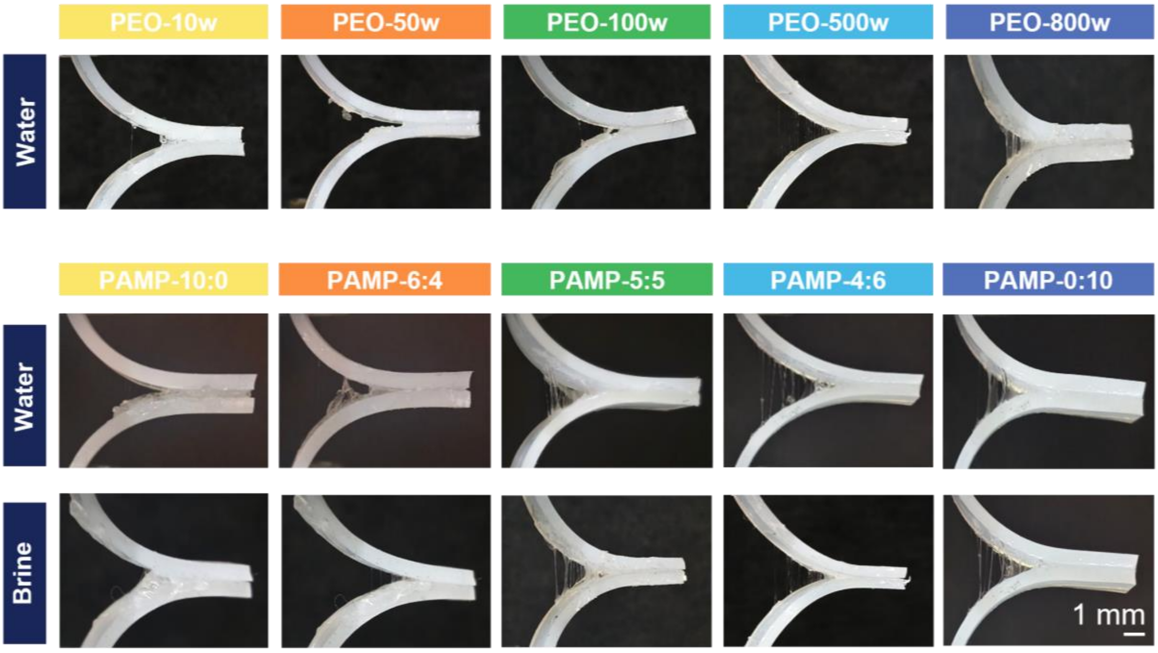

Figure S12. Representative images of 180° peel test for different molecular weights of PEO and different PAM/PEO material constituents of PAMP hydrogel after swelling in either deionized water or 3.6 wt% NaCl solution.

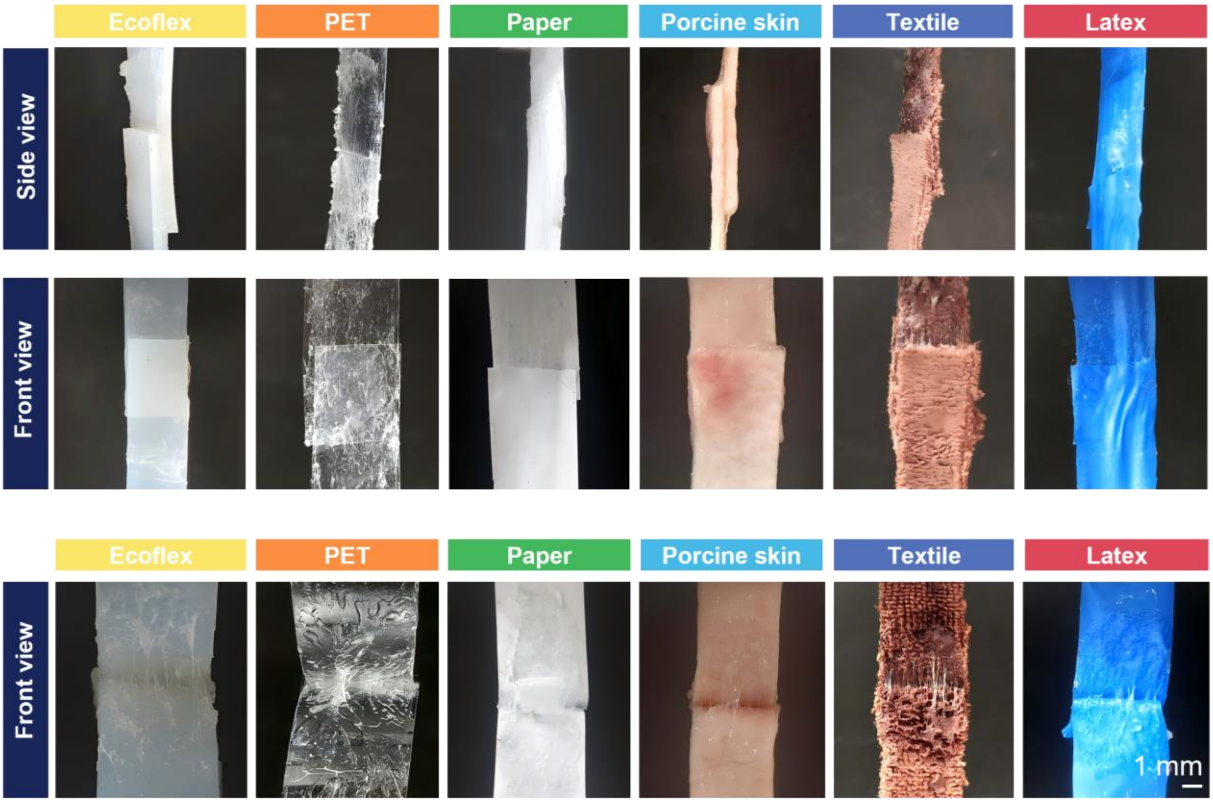

Figure S13. Representative images of lap-shear test and 180° peel test on various substrates after PAMP hydrogel swelling in deionized water.

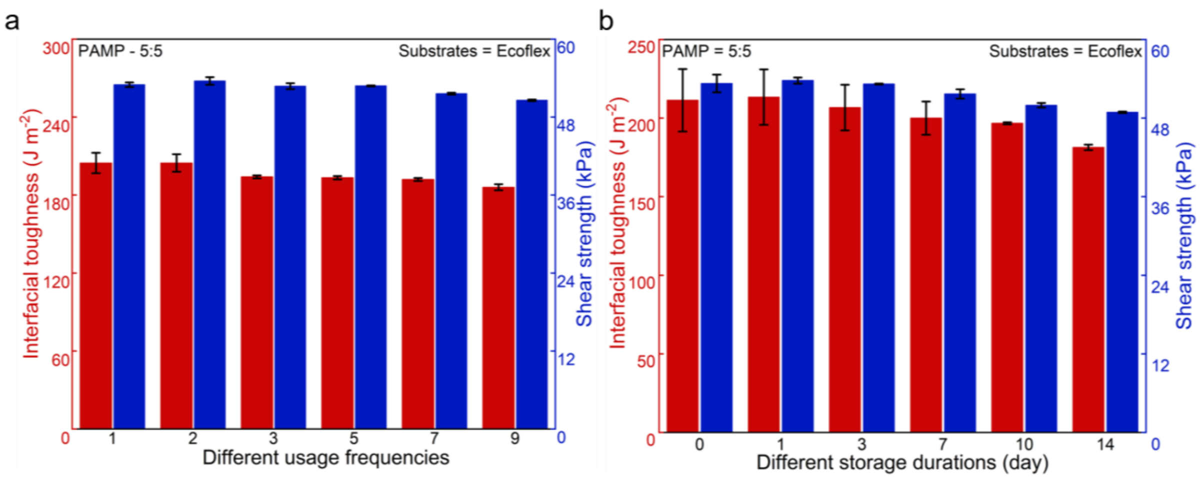

Figure S14. Adhesion performance of PAMP hydrogel. a) Interfacial toughness and shear strength between different usage frequencies. b) Interfacial toughness and shear strength between different storage durations.

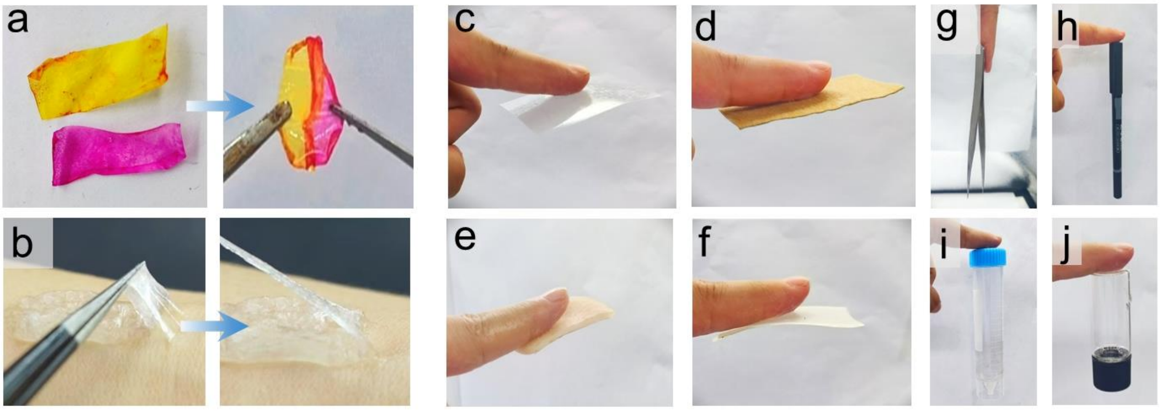

Figure S15. Representative images of adhesion performance. a) Self-healing behavior of the PAMP hydrogel. b) Peeling process demonstrating (left) and (right) low modulus characteristics. c-j. Adhesion to various substrates: c) PET film; d) textile fabric; e) porcine skin; f) Ecoflex silicone; g) metal tweezers; h) ballpoint pen; i) plastic bottle; j) glass bottle.

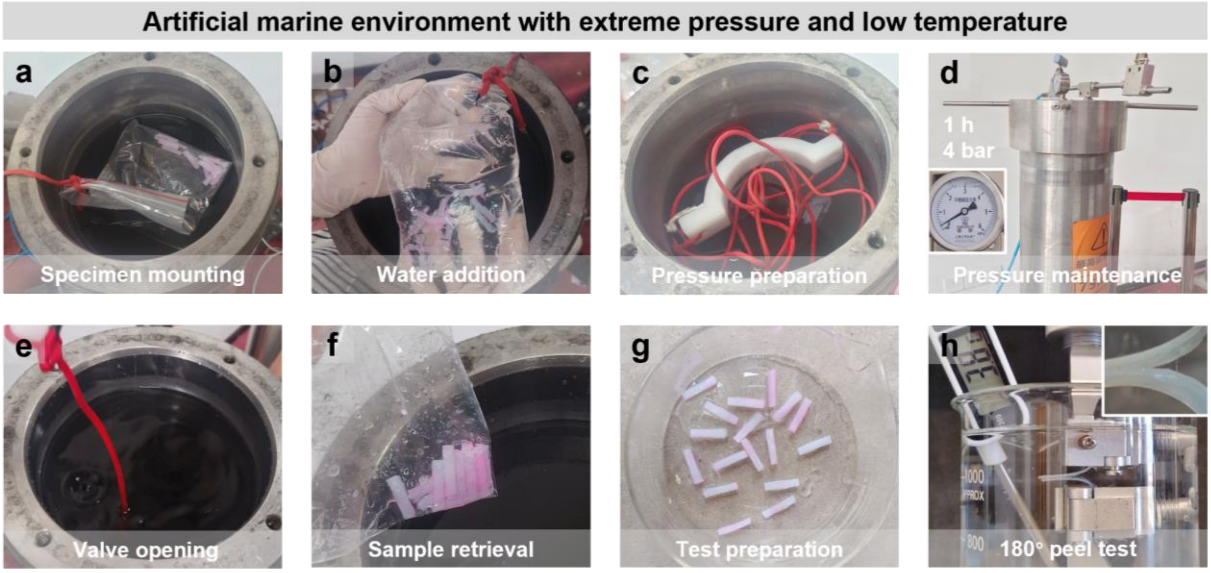

Figure S16. Representative images of PAMP hydrogel underwater adhesion test to simulate the marine environments of pressure and temperature. a) Adhesion specimen (substrate with Ecoflex) mounting in a pressure valve. b) Water addition into the bags. c) Pressure preparation. d) Pressure maintenance (4 bar) for 1 h. e) Valve opening. f) Sample retrieval. g) Adhesion test preparation. h) before and after the hydrostatic pressure underwater 180° peel test.

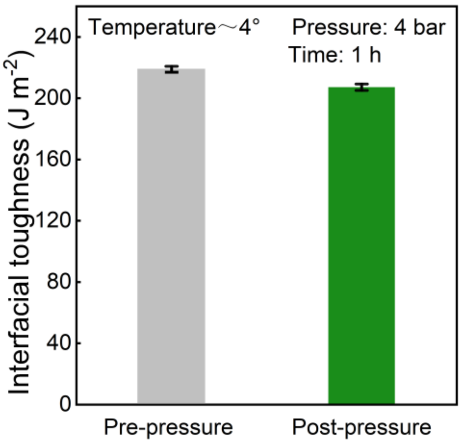

Figure S17. Interfacial toughness between pre-pressure and post-pressure of PAMP hydrogel with the substrate of Ecoflex. (n=15)

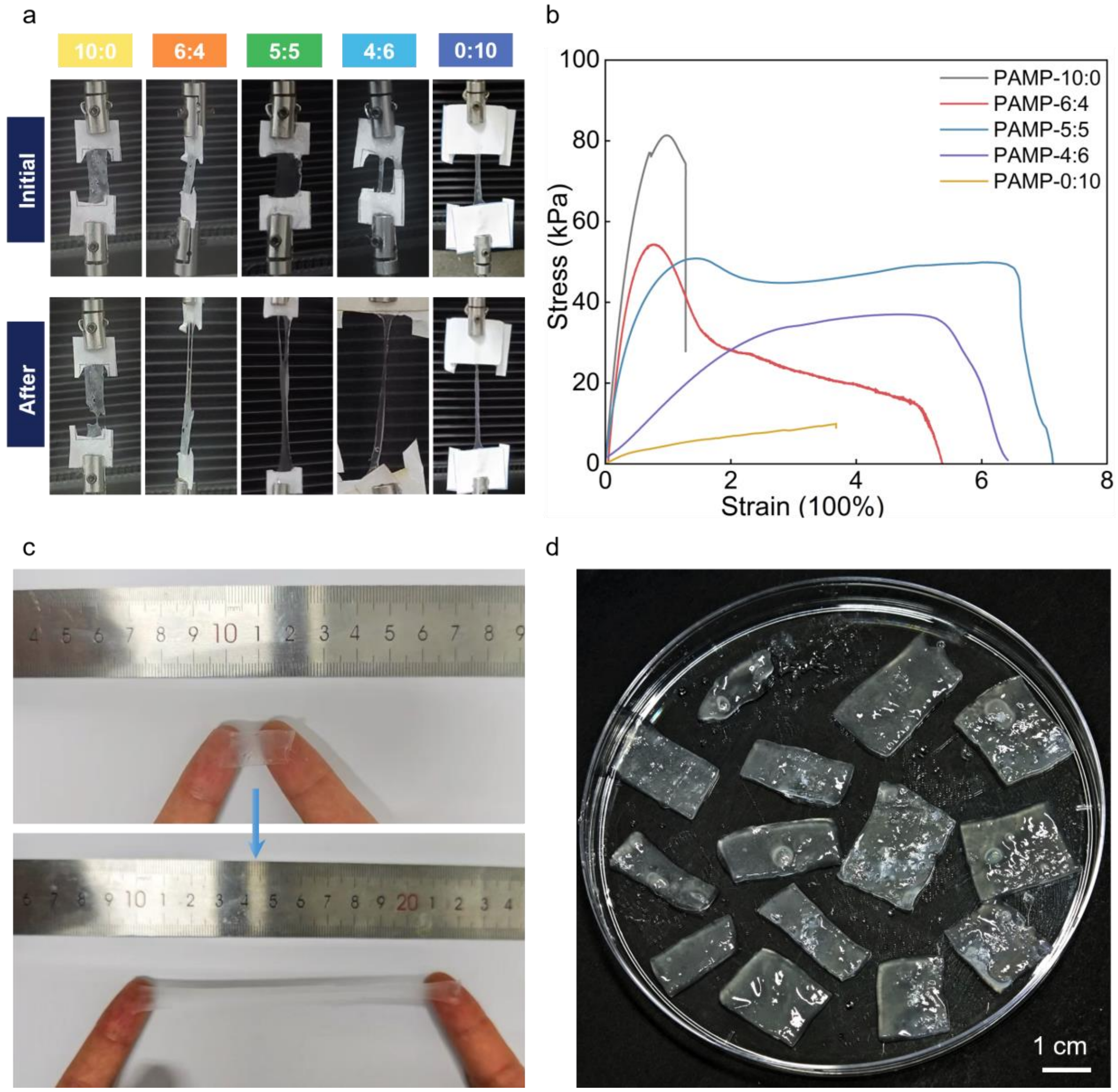

Figure S18. Mechanical performance of PAMP hydrogels. a) Representative of different material constituents, initial and after the stretch test. b) Stress–stretch curves of PAMP hydrogels with varying chemical constituents. c) Excellent tensile properties of PAMP hydrogel. d) Large-scale manufacture for the practical application of PAMP hydrogel.

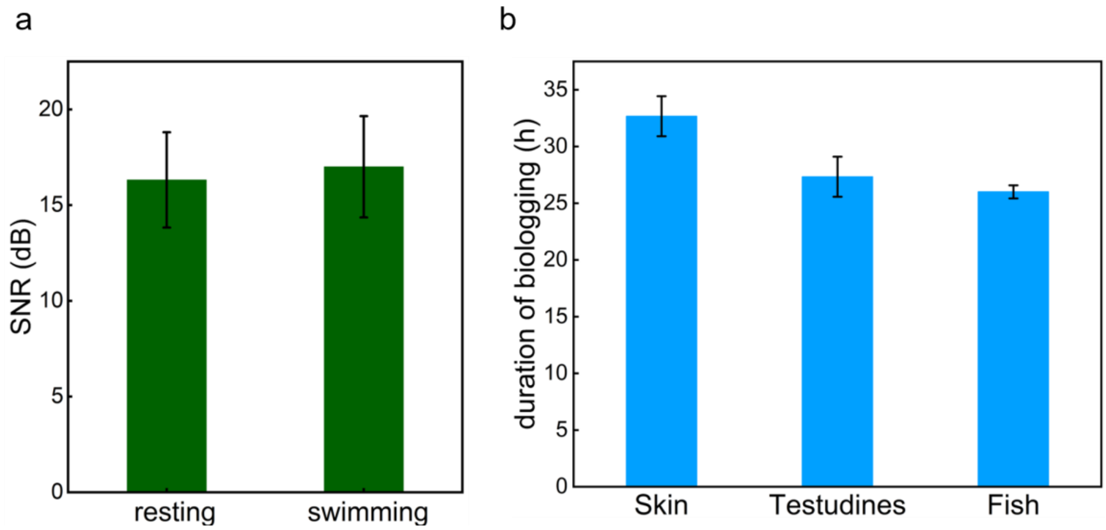

Figure S19. Mechanical properties of PAMP hydrogel. a) Signal-to-noise ratio (SNR) analysis of electrocardiogram (ECG) signals during resting and swimming states. b) Biologging duration comparison across species.

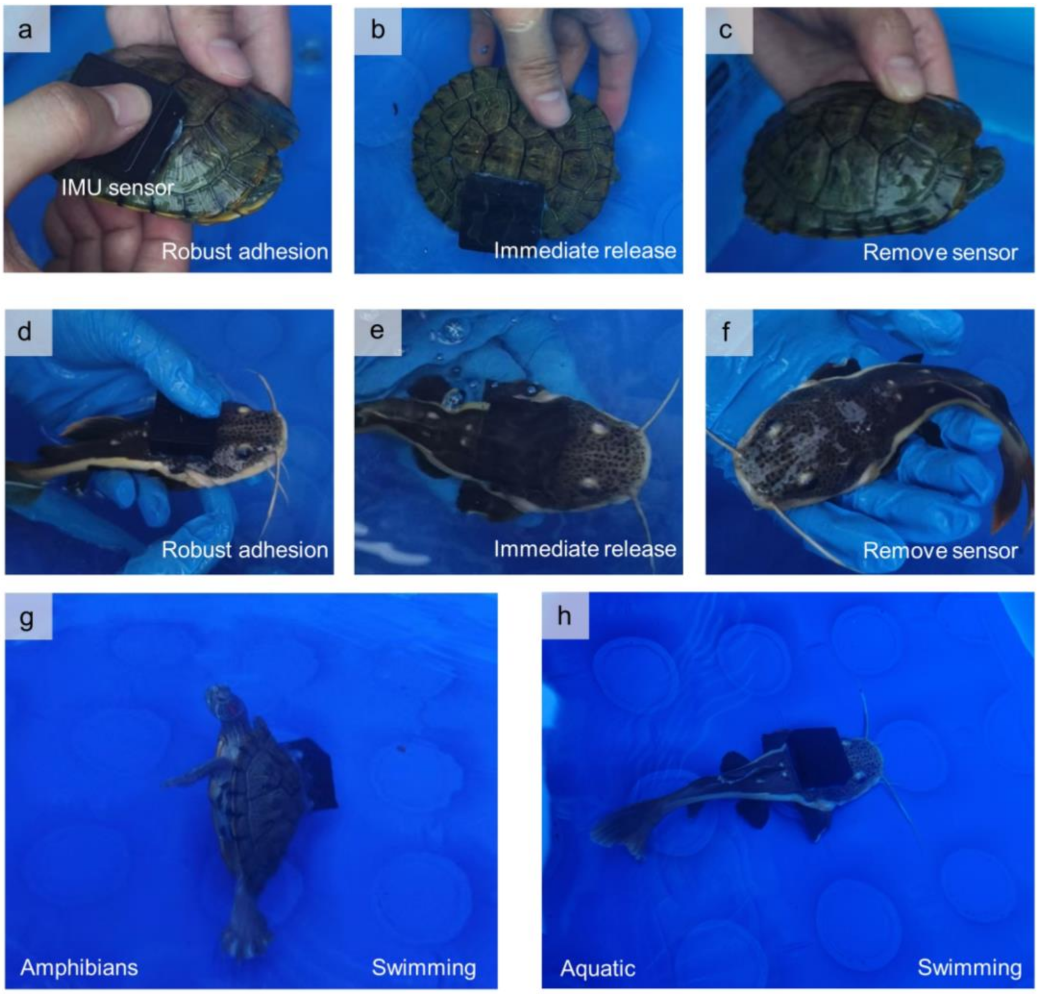

Figure S20. Representative images of movement behaviors monitoring in the pool. a) IMU sensor adhesion on a turtle. b) Releasing the turtle into the water. c) Removing the sensor from the turtle. d) IMU sensor attached to a Silurus asotus. e) Releasing the Silurus asotus into the water; f) Removing the sensor from the Silurus asotus. g) Turtle swimming in the pool. h) Silurus asotus swimming in the pool.

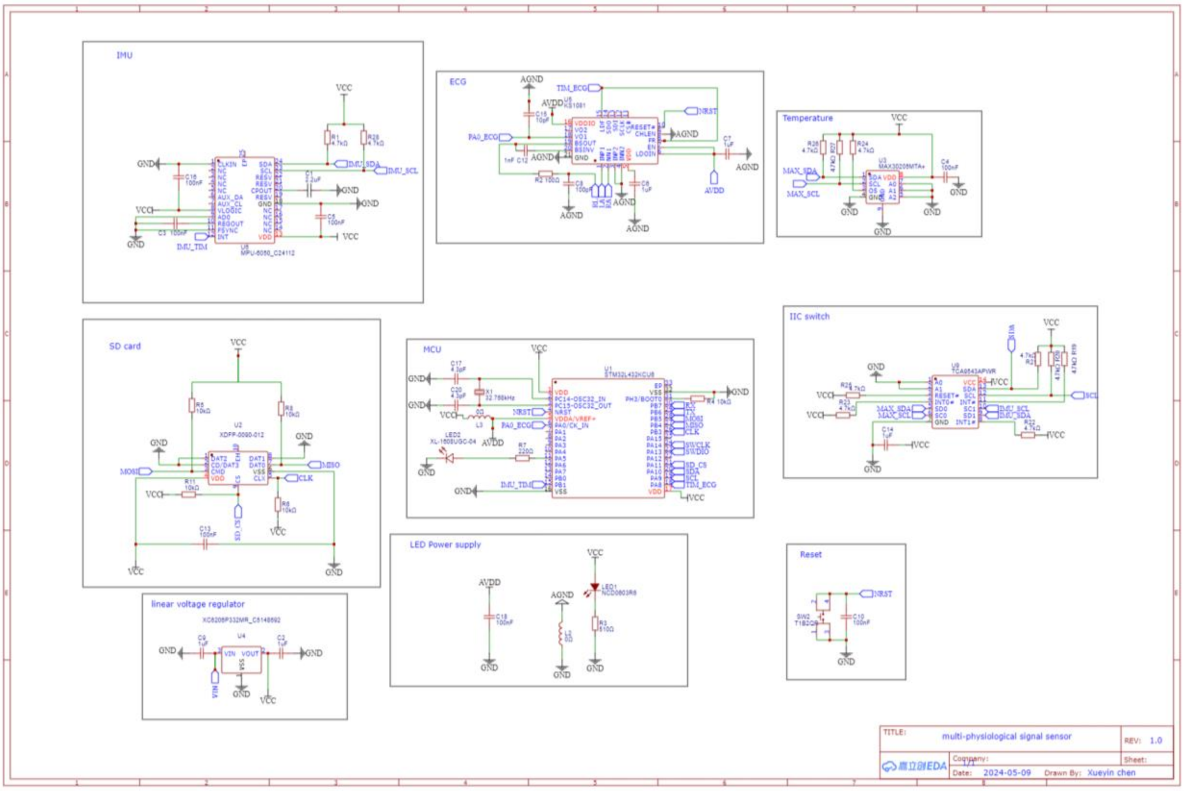

Figure S21. Multi-physiological signal sensor circuit schematic of a flexible multimodal biosensing patch.

Table S1. Mechanical characteristics of PAMP hydrogels with different chemical constituents and PEO molecular weights.

Kind	molecular weight (kDa)	Adhesion time (s)	Interfacial toughness (J m^{-2})	Shear strength (kPa)	Strength (kPa)	Modulus (kPa)	Elongation (%)	Conductivity (10^{-2} S/m)
PAMP -5:5	8,000	15	230	58	50	109	700	3
PAMP -10:0	8,000	358	54	20	83	169	120	0.5
PAMP -6:4	8,000	20	125	48	58	125	550	1
PAMP -4:6	8,000	20	165	49	38	12	650	4
PAMP -0:10	8,000	318	190	53	10	8	390	6.5
PAMP -5:5	100	510	25	8	--	--	--	--
PAMP -5:5	500	358	52	15	--	--	--	--
PAMP -5:5	1,000	240	110	30	--	--	--	--
PAMP -5:5	5,000	60	150	42	--	--	--	--

Table S2. Comparison of various instant wet adhesion hydrogels for biological applications.

Method	Kind	Adhesion time (s)	Interfacial toughness (J m^{-2})	Shear strength (kPa)	Tensile Strength (kPa)	Modulus (kPa)	Elongation (%)	Reference
Interfacial modification	CS/SF/TA	180 hemostasis	--	29.66	158-308	197	200-780	[1]
	PVA/PAA-NHS	22	160	40	--	250	--	[2]
Nanocomposite reinforcement	2D Platelet nanoparticles	600	10	66	1.3	--	8	[3]
	Alg-T/MAP-RGD	180	--	18	--	--	--	[4]
Physical structure engineering	Ultrasound-mediated strategy	60	~1750	40	--	--	--	[5]
	SA/Ca2+/NAGA/AANHS	120	1293	224	205	--	245.69	[6]
	PAMP	15	230	58	50	109	700	This work

References

[1] Z. Qiao, X. Lv, S. He, S. Bai, X. Liu, L. Hou, J. He, D. Tong, R. Ruan, J. Zhang, J. Ding, H. Yang, *Bioactive Materials* **2021**, *6*, *2829*.

[2] C. Duque Londono, S. F. Cones, J. Deng, J. Wu, H. Yuk, D. E. Guza, T. A. Mooney, X. Zhao, *Nat Commun* **2024**, *15*, *2958*.

[3] M. C. Arno, M. Inam, A. C. Weems, Z. Li, A. L. A. Binch, C. I. Platt, S. M. Richardson, J. A. Hoyland, A. P. Dove, R. K. O'Reilly, *Nat Commun* **2020**, *11*, *1420*.

[4] J. Yun, H. T. Woo, S. Lee, H. J. Cha, *Biomaterials* **2025**, *315*, 122948.

[5] Z. Ma, C. Bourquard, Q. Gao, S. Jiang, T. De Iure-Grimmel, R. Huo, X. Li, Z. He, Z. Yang, G. Yang, Y. Wang, E. Lam, Z. Gao, O. Supponen, J. Li, *Science* **2022**, *377*, *751*.

[6] H. Chen, Z. Zhao, R. Zhang, G. Zhang, X. Liang, C. Xu, Y. Sun, Y. Li, C. Boyer, F. Xu, *Advanced Materials* **2025**, *37*, 2413373.

[7] K. E. Sandau, M. Funk, A. Auerbach, G. W. Barsness, K. Blum, M. Cvach, R. Lampert, J. L. May, G. M. McDaniel, M. V. Perez, S. Sendelbach, C. E. Sommargren, P. J. Wang, *Circulation* **2017**, *136*.

[8] P. Rajpurkar, A. Y. Hannun, M. Haghpanahi, C. Bourn, A. Y. Ng, *arXiv preprint arXiv* **2017**, *1707.01836*.

[9] International Electrotechnical Commission, *Medical electrical equipment-Part 2-33* **2010**, *IEC* 60601-2-33 Ed. 3.0.

[10] T. Cui, Y. Qiao, D. Li, X. Huang, L. Yang, A. Yan, Z. Chen, J. Xu, X. Tan, J. Jian, Z. Li, S. Ji, H. Liu, Y. Yang, X. Zhang, T.-L. Ren, *Chemical Engineering Journal* **2023**, *455*, 140690.

[11] M. M. Al Rahhal, Y. Bazi, M. Al Zuair, E. Othman, B. BenJdira, *J. Med. Biol. Eng.* **2018**, *38*, 1014.

[12] D. J. Plews, P. B. Laursen, J. Stanley, A. E. Kilding, M. Buchheit, *Sports Med* **2013**, *43*, 773.

[13] J. Huang, Q. Zhang, T. Zhang, T. Wang, D. Tao, *Sensors* **2024**, *24*, 1041.

[14] M. Buchheit, *Front. Physiol.* **2014**, *5*.

[15] S. K. Nayak, B. Pradhan, B. Mohanty, J. Sivaraman, S. S. Ray, J. Wawrzyniak, M. Jarzębski, K. Pal, *Algorithms* **2023**, *16*, 433.
